# Mechanistic Insights into the Formation of Hydroxyacetone, Acetone, and 1,2-Propanediol from Electrochemical CO_2_ Reduction on Copper

**DOI:** 10.1021/jacs.3c03045

**Published:** 2023-07-10

**Authors:** Alisson H. M. da Silva, Georgios Karaiskakis, Rafaël E. Vos, Marc T. M. Koper

**Affiliations:** Leiden Institute of Chemistry, Leiden University, 2300 RA Leiden, The Netherlands

## Abstract

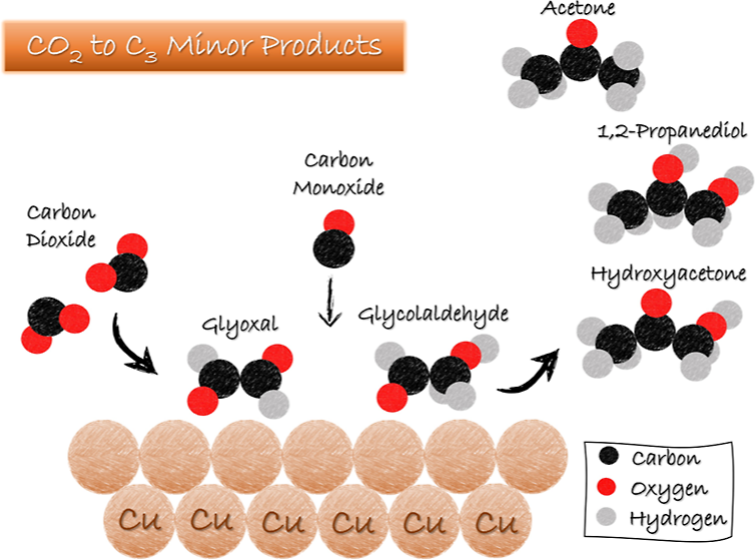

Studies focused on the mechanism of CO_2_ electroreduction (CO_2_RR) aim to open up opportunities to optimize reaction parameters toward selective synthesis of desired products. However, the reaction pathways for C_3_ compound syntheses, especially for minor compounds, remain incompletely understood. In this study, we investigated the formation pathway for hydroxyacetone, acetone, and 1,2-propanediol through CO_(2)_RR, which are minor products that required long electrolysis times to be detected. Our proposed reaction mechanism is based on a systematic investigation of the reduction of several functional groups on a Cu electrode, including aldehydes, ketones, ketonealdehydes, hydroxyls, hydroxycarbonyls, and hydroxydicarbonyls, as well as the coupling between CO and C_2_-dicarbonyl (glyoxal) or C_2_-hydroxycarbonyl (glycolaldehyde). This study allowed us to derive the fundamental principles of the reduction of functional groups on Cu electrodes. Our findings suggest that the formation of ethanol does not follow the glyoxal pathway, as previously suggested but instead likely occurs via the coupling of CH_3_* and CO. For the C_3_ compounds, our results suggest that 1,2-propanediol and acetone follow the hydroxyacetone pathway during CO_2_RR. Hydroxyacetone is likely formed through the coupling of CO and a C_2_-hydroxycarbonyl intermediate, such as a glycolaldehyde-like compound, as confirmed by adding glycolaldehyde to the CO_(2)_-saturated solution. This finding is consistent with CO_2_RR product distribution, as glycolaldehyde formation during CO_2_RR is limited, which, in turn, limits hydroxyacetone production. Our study contributes to a better understanding of the reaction mechanism for hydroxyacetone, acetone, and 1,2-propanediol synthesis from CO_2_RR and gives insights into these interesting compounds that may be formed electrochemically.

## 1 Introduction

CO_2_ electrochemical reduction reaction (CO_2_RR) offers a promising route to store excess renewable electricity in fuels and chemicals. Several of these compounds, including those containing only one carbon in their structures (i.e., C_1_ compounds), such as CO, formic acid, methane, and methanol, as well as compounds with two or more carbons (i.e., C_2+_ compounds), such as ethylene, ethanol, 1-propanol, acetone, and many other oxygenates, can be obtained via CO_2_RR in aqueous media.^1−5^ Copper-based electrocatalysts are known to be the best material to form the C_2+_ backbone for high-value fuels and commodity chemicals.^5−7^ However, the formation of C_2+_ compounds on Cu electrodes is often accompanied by low efficiencies, particularly in the case of C_2+_-oxygenates.

Studies focused on the reaction mechanism provide the opportunity to better understand how a specific compound is formed. This knowledge can be used to design new electrodes and optimize reaction parameters, such as electrode potential and electrolyte composition, to guide the reaction pathway toward the selective synthesis of desired products. For example, {100} facets have been shown to have a better ability to promote CO–CO coupling,^8−10^ which is a key step in the ethylene pathway. Therefore, the use of Cu nanocubes which have {100} nanofacets is a promising strategy for ethylene synthesis, as demonstrated by multiple studies.^11−14^ In the case of 1-propanol, the CO–methylcarbonyl (adsorbed acetaldehyde) coupling is considered a key step for the C_3_ formation.^15−17^ Therefore, developing systems that promote the formation of methylcarbonyl on the electrode surface could lead to a higher faradaic efficiency for 1-propanol.

The reaction mechanism toward the formation of major products such as CO, ethanol, ethylene, and 1-propanol has been extensively investigated by many groups.^18−22^ However, the formation of minor products such as glyoxal, glycolaldehyde, acetone, and hydroxyacetone has not received as much attention. As a result, their formation pathways are not fully understood, especially in the case of the minor C_3_ compounds. Recently, Li et al.^15^ used alkyl iodides to intercept elusive C_1_ and C_2_ intermediates during CORR, providing insights into the reaction pathways of major products such as ethanol, ethylene, and 1-propanol. They have also found insights about acetone formation, one of the minors C_3_-oxygenates, where the coupling between methylcarbonyl and CH_3_* was the suggested likely pathway for its formation. 1-Propanol was mainly formed through methylcarbonyl–CO coupling, consistent with their previous study.^16^ Curiously, the coupling between methylcarbonyl and CO would generate a methyldicarbonyl species (methylglyoxal-like molecule), which can be further reduced to hydroxyacetone and 1,2-propanediol. However, these products were not detected or investigated by the authors. In the same direction, Pablo-García et al.^23^ used experimental and theoretical approaches to investigate the formation mechanism of C_3_ compounds by mixing C_1_–C_2_ molecules and analyzing the outcome when this mixture was reduced at negative potentials. The main C_3_ product detected was 1-propanol, which is primarily formed through methylcarbonyl–CO coupling, as previously shown by Xu’s group.^15,16^ The formation of acetone was also investigated and was attributed to methylcarbonyl–CH_3_* coupling, as also suggested by Li et al.^15^

Hydroxyacetone and acetone exhibit very low faradaic efficiencies during CO_2_RR.^23,24^ Interestingly, acetone can be easily formed on Pd^25^ and Pt^26,27^ electrodes through the reduction of hydroxyacetone. In principle, 1,2-propanediol can also be formed from hydroxyacetone reduction. Therefore, in this work, we have performed a comprehensive study of the pathway for the formation of hydroxyacetone, acetone, and 1,2-propanediol through CO_(2)_RR. Our proposed reaction mechanism is based on a systematic study of the reduction of several functional groups on a Cu electrode, including aldehydes, ketones, ketonealdehydes, hydroxyl, hydroxycarbonyl, and hydroxydicarbonyls, as well as the coupling between CO and C_2_-dicarbonyl (glyoxal) or C_2_-hydroxycarbonyl (glycolaldehyde). Based on the results from the functional group reduction, we could distill several reaction principles on Cu electrodes, such as that aldehydes are preferably reduced over ketones and alcohols need a neighboring carbonyl group to be reduced. Furthermore, our results suggest that hydroxyacetone is most likely formed through the coupling of (dehydrogenated)glycolaldehyde and CO. The presence of glycolaldehyde in the solution results in an increased production of acetone, indicating that it is also formed through the further reduction of hydroxyacetone. Additionally, we indeed observed the formation of 1,2-propanediol, a product which has not been previously reported during CO_(2)_ reduction. Overall, our work gives a (more) complete picture of the pathways, leading to various C_2+_ products during the reduction of CO_2_ on copper electrodes.

## 2 Experimental Section

### 2.1 Chemicals

All electrolytes were made by dissolving appropriate amounts of chemicals in Milli-Q water (Millipore, resistivity ≥ 18.2 MΩ cm). All chemicals were used without any further purification: KOH (99.9%, Sigma-Aldrich), KHCO_3_ (>99.5%, Sigma-Aldrich), K_2_HPO_4_ (99.99%, Merck), KH_2_PO_4_ (99.99%, Merck), KMnO_4_ (ACS reagent, Fluka), H_2_SO_4_ (ACS Reagent, Fluka), H_2_O_2_ (35%, Merck), H_3_PO_4_ (Merck, 85%), formaldehyde (37% in water—contains 10–15% methanol as a stabilizer, Sigma-Aldrich), acetaldehyde (>99.5%, Sigma-Aldrich), propionaldehyde (ACS reagent, Fluka), glyoxal (∼40% in H_2_O, Sigma-Aldrich), acetone (99.5%, Sigma-Aldrich), methanol (99.9%, Merck), ethanol (Absolute, Thermo Fisher Chemical), 1-propanol (99.99%, Sigma-Aldrich), glycolaldehyde dimer (>99.9%, Sigma-Aldrich), ethylene glycol (99.8%, Sigma-Aldrich), 1,2-propanediol (>99.5%, Sigma-Aldrich), 1,3-propanediol (98%, Sigma-Aldrich), glycerol (>99.5%, Sigma-Aldrich), methylglyoxal (∼40% in H_2_O,Merck), hydroxyacetone (95%, Alfa Aesar), dl-2-hydropropanal (∼1 M in H_2_O, Sigma-Aldrich), 3-hydroxypropanal (95%, MolPort), dl-glyceraldehyde (>97%, Sigma-Aldrich), and dihydroxyacetone (97%, Sigma-Aldrich). Gases CO_2_ (Linde, 4.5), CO (Linde, 4.7), and Ar (Linde, 5.0) were used as received.

### 2.2 General Procedures

Prior to each day of experiments, all glassware and the homemade PEEK H-cell were soaked in a 0.5 M H_2_SO_4_ and 1 g/L KMnO_4_ acid solution for at least 12 h. The glassware and H-cell were then rinsed and submerged in a solution of H_2_O_2_ and H_2_SO_4_ to remove any remaining manganese oxide. Next, the solution was drained, and the glassware and H-cell were rinsed with ultrapure water and boiled three times in Milli-Q (≥18.2 MΩ cm) ultrapure water.

In this work, a copper mesh electrode (99.95%, Thermo Scientific Chemicals) with dimensions of 1 cm × 1 cm, mesh number 20, and wire thickness of 0.41 mm was used for all experiments. Before each experiment, the copper mesh was electropolished in 85% H_3_PO_4_ at 2 V for 1 min using a graphite rod as a counter electrode. The electrode was then rinsed with ultrapure water to remove any remaining H_3_PO_4_ solution on the surface.

### 2.3 Electrolysis Tests

In this work, all electrolysis experiments were conducted in a custom-made PEEK H-type cell. A dimensionally stable anode was used as the counter electrode, while a leak-free mini HydroFlex hydrogen electrode (Gaskatel) was used as the reference electrode. The working electrode compartment was separated from the counter electrode compartment using an anion-exchange membrane (Selemion AMVN, AGC). Each compartment was filled with 6 mL of the electrolyte. For the reduction of oxygenates, a 0.1 M potassium phosphate buffer with a pH of 7 was used for all tests. Phosphate buffer was used to avoid too alkaline interfacial pH near the electrode. 0.1 M CO_2_-saturated KHCO_3_ (pH = 6.8) or a 0.1 M KOH (pH = 13) was used as the electrolyte for CO_2_RR and CORR, respectively. In all experiments, the electrolyte was continuously purged with the corresponding gas at a rate of 15 mL/min using mass flow controllers from Brooker. All potentials were controlled with an Ivium potentiostat (Ivium Technologies). Resistances were determined via impedance spectroscopy, and 85% ohmic drop compensation was applied during the experiment. Gas samples were analyzed every 10 min using gas chromatography (Micro-GC, Agilent), equipped with two thermal conductivity detectors (TCDs). One TCD used a CP-SIL 5B column to separate CO_2_, CH_4_, and C_2_H_4_, while the other TCD used a combination of MS5A and CP-PORABOND Q columns to separate H_2_, O_2_, N_2_, CH_4_, and CO. Liquid products were analyzed using high-performance liquid chromatography (Shimadzu) with an Aminex HPX-87H column from BioRad, equipped with two detectors: one refractive index detector and one UV–vis detector with wavenumber set at 205 nm. All faradaic efficiencies, production rates, and error bars reported in this work were calculated by calculating the average of (at least) three replicates of each studied potential.

The ^1^H-NRM analysis was carried out in a Ascend 600 Bruker spectrometer using 600 MHz of frequency. Typically, 540 μL of the sample was mixed with 60 μL of D_2_O solution containing 5 mM phenol as an internal standard. The spectra were collected with 16 s relaxation time between the pulses to allow for complete proton relaxation. The water suppression mode was used.

## 3 Results and Discussion

The reaction pathway leading to C_3_ compounds from CO_2_RR involves several steps, including C–C coupling, formation of hydroxyls, ketones, aldehydes, hydrocarbons, and mixtures of these functional groups, such as hydroxyketones and hydroxyaldehydes. Therefore, understanding how these groups behave under reducing potentials on Cu electrodes is a crucial step in identifying the most likely intermediates in the reaction mechanism for C_3_ compounds from CO_2_RR. To achieve this, we have dedicated five sections in the Supporting Information (Sections S1–S5) to study the electroreduction of these functional groups on Cu electrodes. Specifically, Section S1 focuses on the electroreduction of aldehydes, Section S2 on alcohols, Section S3 on ketones, Section S4 on ketone aldehydes, and Section S5 on hydroxycarbonyls. The results and discussion provided in these sections form the basis for the reaction mechanism of C_3_ compounds from CO_2_RR proposed in this work.

**Figure 1:**
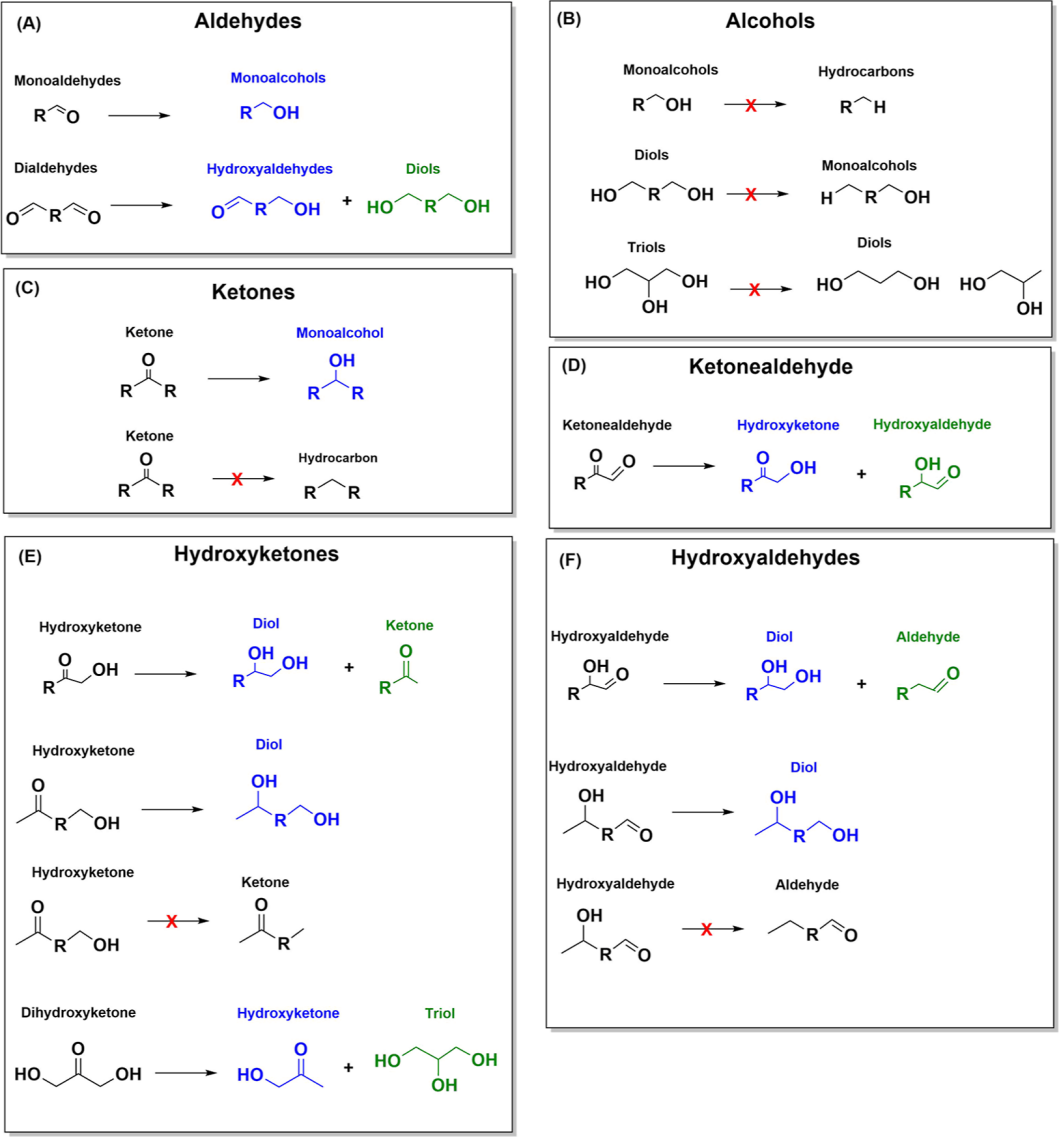
Primary products on a Cu electrode at neutral pH generated upon reducing (a) aldehydes; (b) alcohols; (c) ketones; (d) ketonealdehhyde; (e) hydroxyketones; and (f) hydroxyaldehydes. Products in blue represent the preferred compound; in green represent the products less favorably formed. Products in black are unlikely to be formed in neutral pH on the Cu electrode (as also indicated by the arrow with the red cross).

All results present in Sections S1–S5 were carried out in the phosphate buffer electrolyte (pH 7) to prevent base-promoted homogeneous reactions and conduct these tests in a pH range similar to that typically employed for CO_2_RR (pH 6.8), but in the absence of CO_2_ to prevent C–C coupling with CO_(2)_* species under reductive potentials. In summary, Section S1 shows that monoaldehydes are only reduced to their corresponding monoalcohols, and no hydrocarbons are formed, indicating that the oxygen atom in the carbonyl group is not removed from a monoaldehyde. Dialdehydes are primarily reduced to their hydroxyaldehydes, but diols, monoaldehydes, and monoalcohols were also detected. In dialdehydes, one of the oxygen atoms from the carbonyl group could be removed to form the monoaldehyde and subsequently reduced to the corresponding monoalcohol, but the second oxygen containing functional group remained intact as hydrocarbons were not detected. Section S2 reveals that alcohols cannot be further reduced to hydrocarbons, irrespective of the number of carbons in the carbon chain, the position of the hydroxyl, or the number of hydroxyls in the molecule. A hydroxyl group can only be reduced if a carbonyl group is adjacent, as shown in Section S5. Similarly, like monoaldehydes, Section S3 indicates that acetone (the monoketone molecule evaluated) was solely reduced to 2-propanol, though with minimal reactivity. The location of the carbonyl in the carbon chain plays a crucial role as the reduction of propionaldehyde shows significantly higher yields. In Section S4, the reduction of ketonealdehydes was investigated, using methylglyoxal as a model molecule. At lower overpotentials, the primary products observed were hydroxycarbonyls (hydroxyacetone and 2-hydroxypropanal). Hydroxyacetone was preferred over 2-hydroxypropanal, due to the higher reactivity of the aldehyde group, as demonstrated by the reduction of propionaldehyde compared to acetone. Although the aldehyde group is more reactive than the ketone group, the presence of the aldehyde group adjacent to the ketone significantly enhanced the reactivity of the ketone. At higher overpotentials, the ketonealdehydes can be further reduced to form alcohols, diols, and aldehydes. In Section S5, we have systematically investigated the behavior of the hydroxyl group in the presence of an adjacent carbonyl group. As a general trend, our results suggest that at lower overpotentials, the most favored reaction is the reduction of the carbonyl group to its corresponding alcohol. At potentials more negative than −0.8 V, it becomes more apparent that the hydroxyl group adjacent to the carbonyl group can be removed. Dihydroxyacetone was the exception for this trend, for which hydroxyacetone was favored over glycerol in the entire potential range evaluated. However, if the hydroxyl group is not adjacent to the carbonyl group, it remains unchanged. For instance, when hydroxyacetone, a molecule that contains a carbonyl adjacent to the hydroxyl, was reduced, 1,2-propanediol and acetone were detected. However when 3-hydroxypropanal, a molecule in which the carbonyl is not adjacent to the hydroxyl, was reduced, only 1,3-propanediol was formed, and no propionaldehyde was identified. A summary of the primary products generated upon reducing oxygenates on a Cu electrode at neutral pH presented in Sections S1–S5 is shown in Figure 1. The preferred product from the reduction of a specific group is represented in blue, while the products which are formed less favorably are represented in green. Products that are unlikely to form on Cu under conventional CO_2_RR conditions are illustrated in black and an arrow with a red cross. This summary serves as the fundamental basis for the reaction mechanism proposed in this study toward C_2_ and C_3_ compounds.

**Figure 2:**
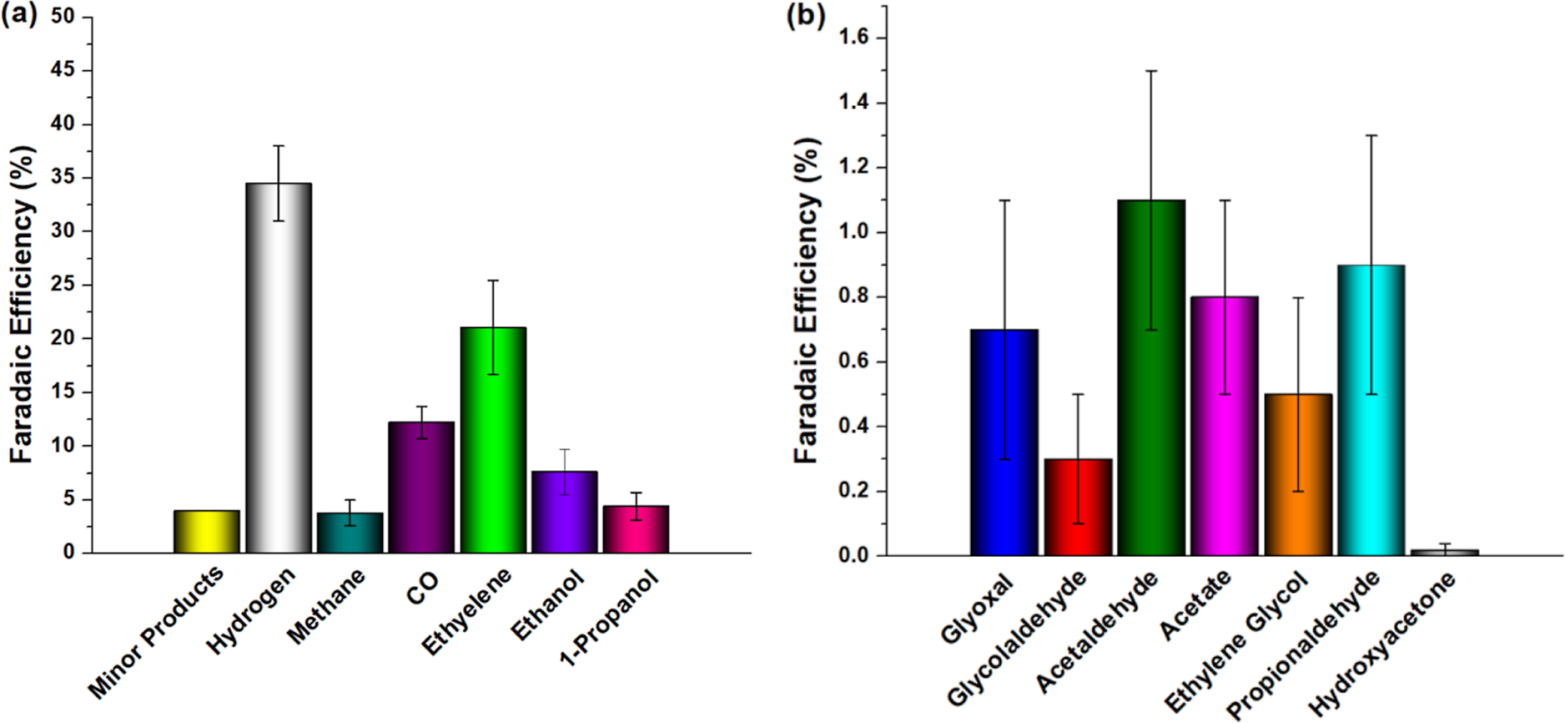
Faradaic efficiencies for (a) major products: hydrogen, methane, CO, ethylene, ethanol, and 1-propanol; and for (b) minor products: glyoxal; glycolaldehyde; acetaldehyde; acetaldehyde; ethylene glycol; propionaldehyde; and hydroxyacetone from CO_2_RR in the CO_2_-saturated 0.1 M KHCO_3_ electrolyte at −1.0 V vs RHE.

**Figure 3:**
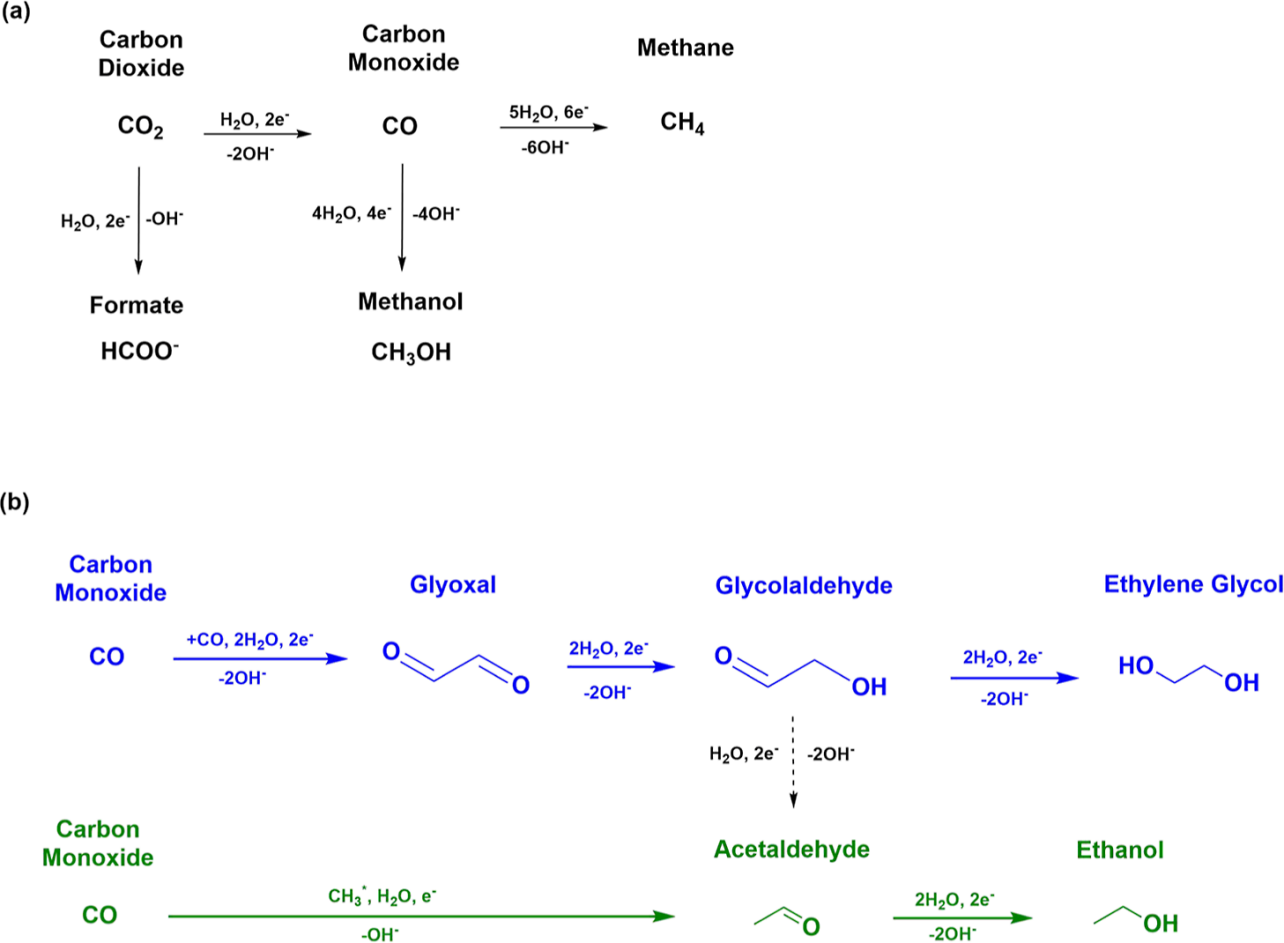
Reaction pathway for (a) C_1_ and (b) C_2_ liquid products from CO_2_RR on the Cu electrode. The blue mechanism in (b) represent the likely pathway for ethylene glycol, glycolaldehyde, and glyoxal based on the results in this work, as also proposed by Garza et.al.^22^ The pathway in green is based on the results shown by Li et al.^15^ and the trends observed in this work and the work of Delmo et al.^30^

Figure 2 displays the faradaic efficiency for H_2_, CO, methane, formic acid, ethylene, ethanol, acetate, glyoxal, glycolaldehyde, ethylene glycol, propionaldehyde, and 1-propanol from CO_2_ reduction on a Cu electrode after transferring 30C in a CO_2_-saturated KHCO_3_ electrolyte at −1.0 V vs RHE. For the formation of C_1_ products, formic acid is formed from the reduction of CO_2_ to HCOO– that will be further protonated to HCOOH in acidic media. CO is generated by reducing CO_2_ in a two-electron transfer step, releasing two hydroxyl anions per CO molecule formed (CO_2_ + H_2_O + 2e^–^ → CO + 2OH^–^).^28^ Subsequently, CO can be further reduced to CH_4_ in a six-electron transfer step. Methanol was not measured in this study, although a low amount has been observed from CO reduction on the Cu electrode.^4,24^ The summary of C_1_ compound formation is presented in Figure 3a, but more detailed information can be found elsewhere in the literature.^18−22,29^ Liquid C_2_ compounds have been reported to be formed through CO–CO coupling,^5,18,22,29^ which subsequently undergo reduction to yield the corresponding aldehydes (glyoxal or acetaldehyde), hydroxyaldehyde (glycolaldehyde), or alcohols (ethylene glycol or ethanol). Based on the results obtained from Sections S1–S5 and summarized in Figure 1, glycolaldehyde is formed through the reduction of glyoxal. Furthermore, glycolaldehyde is further reduced to produce ethylene glycol and acetaldehyde (as shown in Figure S7a). In turn, acetaldehyde can be reduced to form ethanol. This pathway involving glyoxal, glycolaldehyde, ethylene glycol, acetaldehyde, and ethanol has already been predicted from DFT calculations by Garza et al.^22^ and shown experimentally by Schouten et al.^29^ The faradaic efficiencies of glyoxal, glycolaldehyde, and ethylene glycol, as shown in Figure 2, are consistent with the prediction made by Garza et al. These products exhibit similar productivities and follow the expected trend, with ethylene glycol presenting a higher faradaic efficiency than glycolaldehyde at −1.0 V, as observed in Figure S3 during the reduction of glyoxal. However, if we consider that ethanol also follows the glyoxal pathway, as shown by Garza et al. and Schouten et al., the results obtained for the reduction of glycolaldehyde and ethylene glycol should exhibit at least similar or higher faradaic efficiencies than ethanol, considering the fundamental principles illustrated in Figure 1, as well as the results obtained from the reduction of glycolaldehyde (Figure S7a). During glyoxal reduction (Figure S3), ethylene glycol was preferred over ethanol, even at potentials more negative than −1.0 V. During glycolaldehyde reduction (Figure S7a), similar faradaic efficiencies were observed for ethylene glycol and ethanol at potentials more negative than −0.9 V. Therefore, these results are not consistent with the faradaic efficiency obtained after 30C of CO_2_RR, where ethanol exhibited faradaic efficiencies ∼20 times higher than ethylene glycol and glycolaldehyde. This suggests that ethanol does not (solely) follow the glyoxal and glycolaldehyde pathways, as observed for ethylene glycol. Recently, Delmo et al.^30^ also evaluated the selectivity of ethylene glycol and ethanol from glyoxal reduction, and they have also concluded that glyoxal may not be the main intermediate toward ethanol production in CO_2_RR on Cu. Li et al.^15^ demonstrated that ethanol could be selectively formed via CH_3_* and CO coupling using alkyl intermediates. Additionally, it is worth noting that CH_4_ is commonly produced at −0.9 V vs RHE in a CO_2_-saturated KHCO_3_ electrolyte, indicating the presence of CH_x_* species from this potential. For example, Kuhl et al.,^24^ Singh et al.,^31^ and Hori et al.^7^ demonstrated that ethanol formation is favored under reaction conditions where methane production is also prominent. This correlation suggests that when ethanol is formed, CH_3_* and/or CH_x_* species are likely present. Therefore, our results and understanding suggest that ethanol formation may follow different pathways. In addition to the well-known glyoxal pathway, ethanol may also be formed through the coupling of CH_x_* intermediates with *CO in an alternative pathway. It is also worth mentioning that the current density measured for the tests, as shown in Sections S1–S5 (in phosphate buffer electrolyte), was found to be around −10 mA/cm^2^, while for CO_2_RR in the CO_2_-saturated electrolyte KHCO_3_ (Figure 2), the current density was around −7 mA/cm^2^. Therefore, we assume that the possible pH difference does not invalidate the trends observed in the reaction mechanism we have proposed. Additionally, we have recently shown that the addition of buffer (phosphate is this case) is the most suitable strategy to suppress interfacial pH gradients.^32^

The formation of ethylene, the main C_2_ product on Cu electrodes, appears to be an exception to the principles shown in Figure 1. The ethylene reaction pathway has been extensively studied, and it is reported to be formed from CO–CO coupling, which can be reduced to O=CH–C=O* and further converted to C_2_H_4_.^18,33,34^ Therefore, both carbonyl groups could be further reduced to their corresponding hydrocarbons, which was not observed in any other molecule evaluated in Sections S1–S5. It is worth mentioning that the principles summarized in Figure 1 were developed by studying the reduction of liquid compounds, so it is possible that the rules may differ for gases or that a different reaction pathway is followed for ethylene. Figure 3b provides a summary of the reaction pathways for the formation of glyoxal, glycolaldehyde, and ethylene glycol (in blue), and for acetaldehyde and ethanol (in green), with the latter based on the results reported by Li et al. and the trends observed in this work and in the work of Delmo et al.

**Figure 4:**
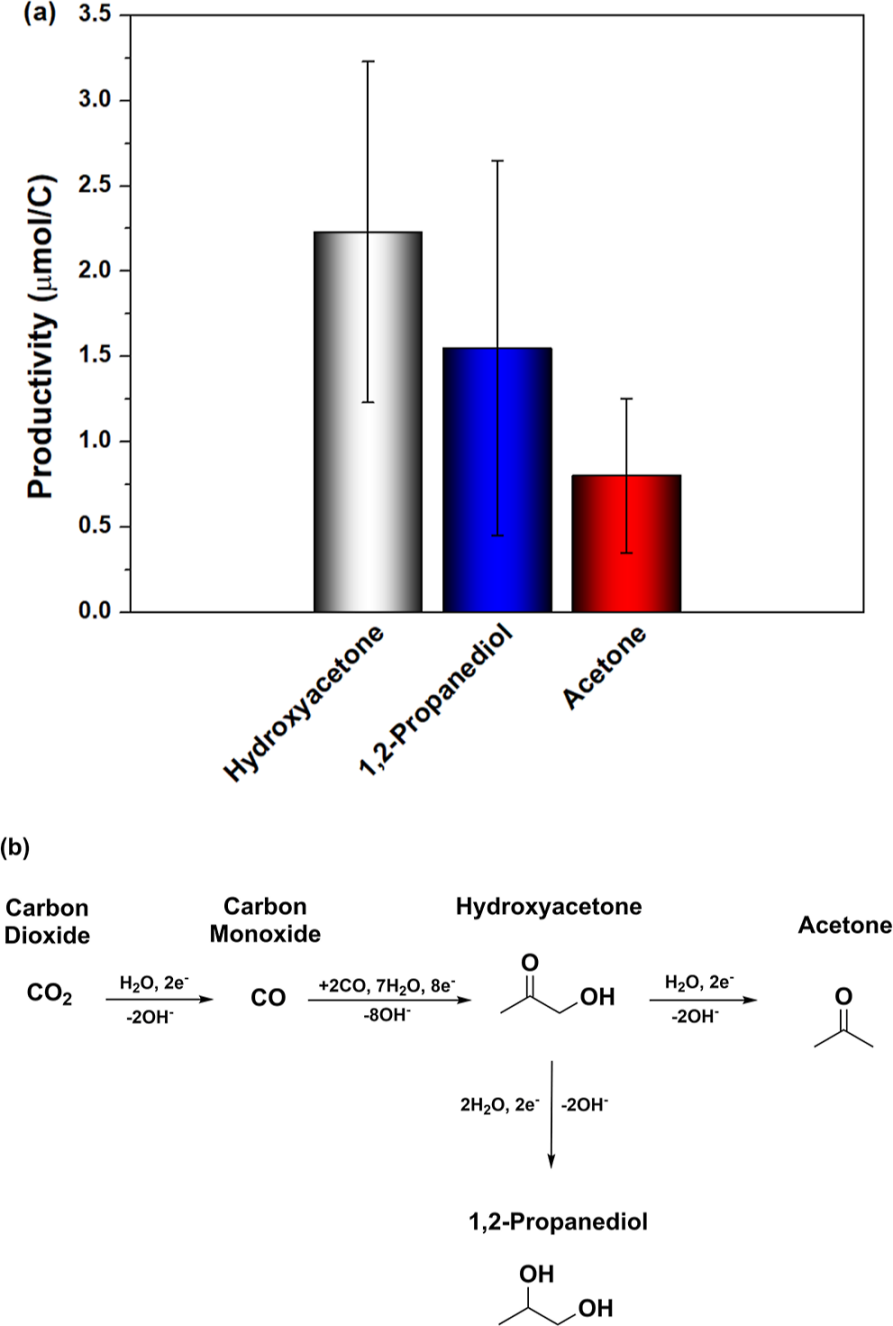
(a) Production of hydroxyacetone (gray bar), 1,2-propanediol (blue bar), and acetone (red bar) in CO_(2)_-saturated 0.1 M KHCO_3_ electrolyte after 200C of charge was transferred. (b) Reaction pathway from CO_2_ to hydroxyacetone, acetone, and 1,2-propanediol.

For C_3_ compounds, 1-propanol is the predominant product. 1-Propanol is known to be produced through the coupling of adsorbed methylcarbonyl and CO, which leads to the formation of propionaldehyde and subsequently reduced to 1-propanol.^15−17^ The experimental insights suggesting methylcarbonyl as a probable intermediate leading to 1-propanol were obtained by introducing a stable C_2_ compound, namely, acetaldehyde, into the electrolyte and observing the resulting products after electrolysis. This approach is a useful strategy to experimentally identify potential intermediates, although it does not rule out the possibility of alternative C_2_ intermediates participating in the reaction pathway. The reaction pathway for 1-propanol will not be discussed here as it has been discussed in detail elsewhere.^15−17^ Additionally, a small amount of hydroxyacetone (FE < 0.05%) was detected, which was also observed by Kuhl et al.^24^ However, unlike 1-propanol, the reaction mechanism for hydroxyacetone formation has not yet been investigated. Interestingly, based on the reduction of hydroxycarbonyls, as shown in Figure S7, as hydroxyacetone was detected, it is also expected to form 1,2-propanediol and acetone since its reduction leads to these molecules at −1.0 V. Nonetheless, it is possible that these products were formed, but their productivity was below the chromatograph’s detection limit. To enhance the concentration of minor products in the solution, a mixture of CO_2_ and CO (CO_2_/CO = 4:1, v/v) was reduced in 0.1 M KHCO_3_ electrolyte at −1.0 V until a total charge of 200C was transferred, and the results are shown in Figure 4a. The CO_2_/CO mixture was used to enhance CO–CO coupling by introducing CO in the inlet stream, while continuously bubbling CO_2_ into the electrolyte to maintain a neutral pH electrolyte. The pH was measured to be 7.2 after gas saturation. Faradaic efficiencies were not determined due to difficulties in identifying the source (CO_2_ vs CO) for the synthesis. In contrast to Figure 2, Figure 4a shows the formation of acetone. Acetone has been identified as a minor product from CO_2_RR before.^23,29^ In addition to acetone, 1,2-propanediol was also identified as a product and confirmed by H^1^-NMR analysis, as shown in Figure S8 and described in Note S1 in the Supporting Information. To the best of our knowledge, we show here for the first time the formation of 1,2-propanediol from CO_(2)_RR. 2-Propanol was not identified, possibly due to the reduction of acetone being practically inactive on Cu in neutral pH (Figure S4). The higher formation of 1,2-propanediol compared to acetone is consistent with the principles illustrated in Figure 1 and the results shown in Figure S7b for hydroxyacetone reduction. Thus, here we also show an alternative pathway for acetone formation besides the coupling between methylcarbonyl and CH_3_*, as predicted by Li et al.^15^ and Pablo-García et al.:^23^ both 1,2-propanediol and acetone follow the hydroxyacetone pathway, as illustrated in Figure 4b.

**Figure 5:**
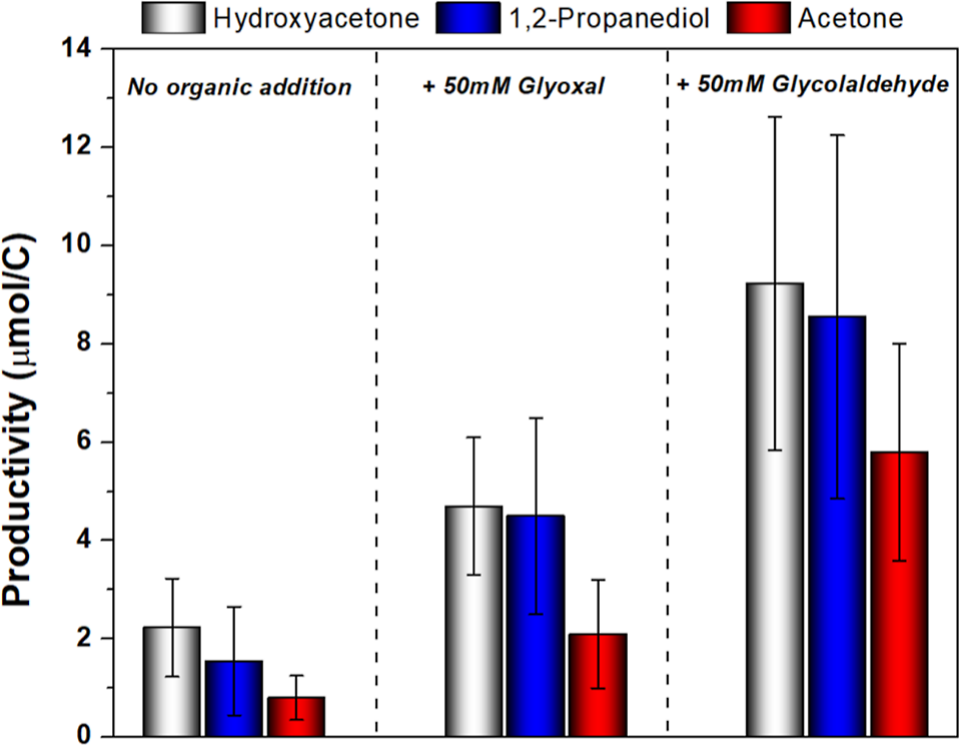
Production of hydroxyacetone (gray bar), 1,2-propanediol (blue bar), and acetone (red bar) in CO_(2)_-saturated 0.1 M KHCO_3_ electrolyte containing 50 mM of glycolaldehyde after 200C of charge was transferred.

Although Figure 4b shows that the reduction of hydroxyacetone results in the formation of 1,2-propanediol and acetone, the mechanism for the formation of hydroxyacetone is unclear. It is likely that hydroxyacetone has an unfavorable intermediate since its formation rate is rather low. For example, ethylene glycol and glycolaldehyde present lower FEs because their intermediate glyoxal is not preferably formed under standard CO_2_RR conditions. Ethanol and 1-propanol are major products presented in Figure 2, and both alcohols are reported to have methylcarbonyl as an intermediate.^15,23,35^ In turn, adsorbed methylcarbonyl is identified as a common C_2_ intermediate formed on Cu electrodes,^15^ which explains why ethanol and 1-propanol can have high productivities. In other words, the productivity of a specific compound depends on how easily its key precursor intermediate is formed under the reaction conditions applied. Similar to what happens with 1-propanol synthesis, where CO couples with a C_2_-monocarbonyl intermediate to selectively form a C_3_-monoaldelhyde which is further reduced to its C_3_-monoalcohol, a C_2_-dicarbonyl or C_2_-hydroxycarbonyl intermediate is likely required to couple with CO and form a C_3_-hydroxyketone (hydroxyacetone). Therefore, glyoxal and/or glycolaldehyde are likely the C_2_-dicarbonyl and/or C_2_-hydroxycarbonyl intermediates for hydroxyacetone formation. The formation rates of glyoxal and glycolaldehyde shown in Figure 2 support this hypothesis since the formation of glycolaldehyde and glyoxal is relatively low and, therefore, the formation of hydroxyacetone is expected to be even lower. To verify this hypothesis, a mixture of CO_2_ and CO (CO_2_/CO = 4:1, v/v) was reduced in 0.1 M KHCO_3_ electrolyte containing 50 mM of glyoxal or glycolaldehyde at −1.0 V until a total charge of 200C was transferred. The formation of hydroxyacetone, 1,2-propanediol, and acetone was higher when glyoxal or glycolaldehyde was added to the solution than when the electrolysis was carried out in the absence of these compounds, as shown in Figure 5. This strongly indicates that the C_2_-dicarbonyl and/or C_2_-hydroxycarbonyl are likely intermediates for hydroxyacetone formation. Acetaldehyde was also tested under the same reaction conditions in order to investigate whether methylcarbonyl could also be a C_2_ intermediate for hydroxyacetone, but neither hydroxyacetone nor 1,2-propanediol and acetone were detected. Only 1-propanol was observed as C_3_ compound, as also observed before.^15,23,35^ The formation of hydroxyacetone and 1,2-propanediol was found to be higher in the electrolyte that contained glycolaldehyde compared to the one containing glyoxal. This observation suggests that glycolaldehyde is the most likely intermediate for hydroxyacetone formation. However, we do not have enough insights at this moment to confirm whether hydroxyacetone was enhanced in the presence of glyoxal due to its further reduction to glycolaldehyde and subsequent coupling with CO, or if both molecules can undergo C–C coupling with CO, but glycolaldehyde–CO coupling forms hydroxyacetone more selectively.

The production of hydroxyacetone, 1,2-propanediol, and acetone is enhanced in the presence of glycolaldehyde. However, the production is still rather low even when the electrolyte contains 50 mM of the presumed intermediate. We faced a similar problem when we used acetaldehyde as an intermediate to enhance 1-propanol formation.^17^ Acetaldehyde is not the actual intermediate for 1-propanol synthesis but dehydrogenated acetaldehyde (or adsorbed methylcarbonyl). It is challenging and thermodynamically unfavorable to dehydrogenate acetaldehyde under reducing potentials in a neutral pH electrolyte, which explains why the production of 1-propanol is enhanced only to a limited extent when millimoles of acetaldehyde are added to the electrolyte. Similarly, glycolaldehyde is not the intermediate for hydroxyacetone formation but likely the adsorbed dehydrogenated glycolaldehyde. Thus, in order to enhance the formation of dehydrogenated glycolaldehyde and increase the production of C_3_ minor products, CORR was carried out in 0.1 M KOH with interval addition of glycolaldehyde to the electrolyte, and the results are shown Figure S9. More experimental details can be found at Note S2 in the Supporting Information. Our results showed that acetone was not observed, likely due to its aldol dimerization in alkaline media.^36^ 1,2-Propanediol and hydroxyacetone increased as more acetaldehyde was added to the system. When no glycolaldehyde was added, there was no increase in the productivity of hydroxyacetone, and a small decrease was actually observed. Conversely, 1,2-propanediol increased slightly, indicating that hydroxyacetone was partially reduced to 1,2-propanediol as expected from the results shown in Figure S7b.

**Figure 6:**
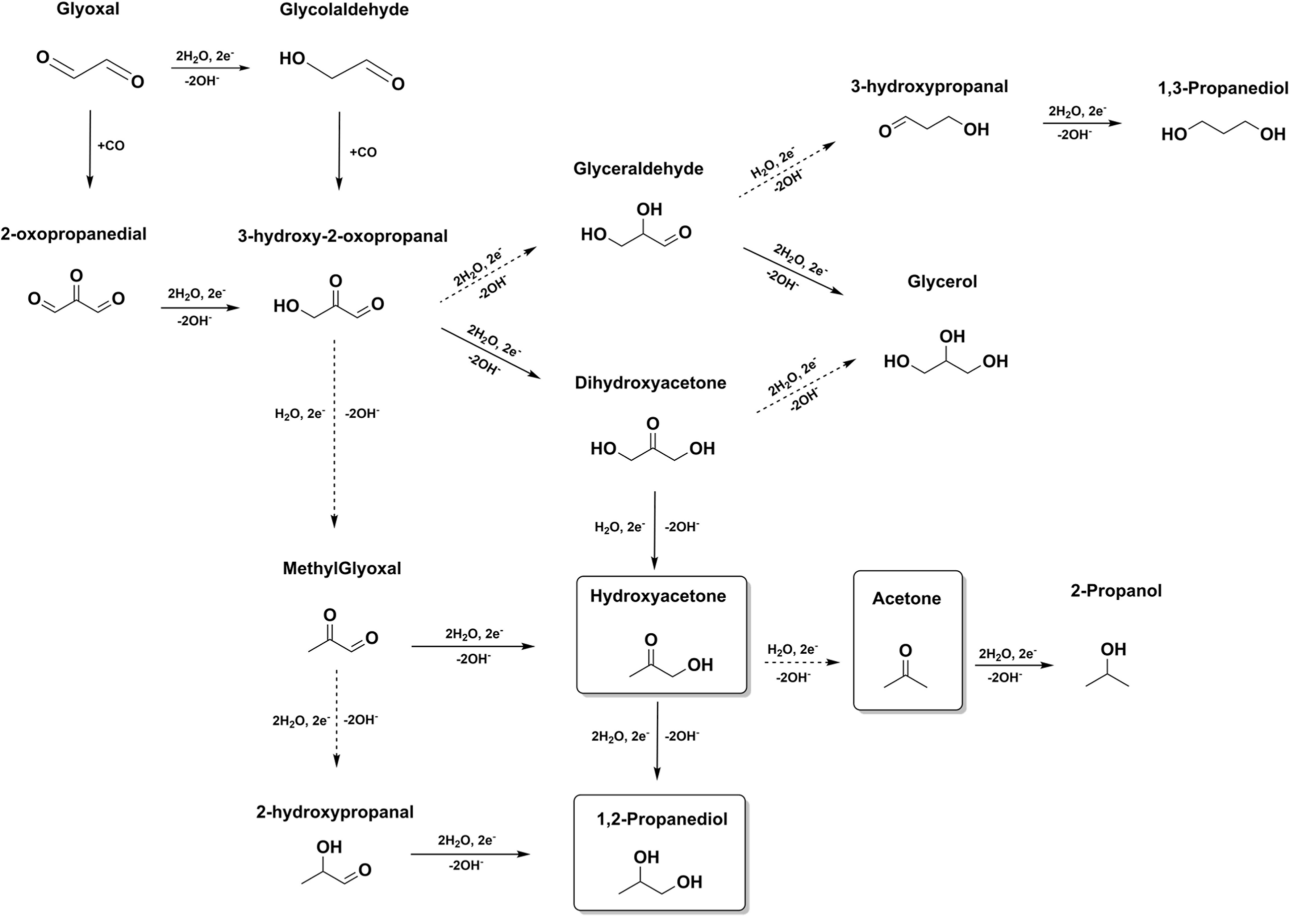
Reaction pathways from glyoxal and glycolaldehyde to C_3_ minor products. The full line arrows indicate the preferred reduction products, while the dashed line arrows represent the less preferred ones. Square blocks represent the products identified from CO_2_RR. The other C_3_ products are based on the results presented in Sections S1–S5 but not identified from CO_(2)_RR.

Figure 6 shows an extended overview of the suggested pathways including also C_3_ intermediates. The overview is developed based on the principles summarized in Figure 1, along with the results from Figures 4, 5, and S9, which demonstrate the CO–glycolaldehyde coupling. The full line arrows in the figure indicate the preferred reduced compounds, while the dashed line arrows represent the less preferred reduction products. Sections S3 and S4 demonstrate that the aldehyde group is more reactive than the ketone group, and thus, dihydroxyacetone is preferred over glyceraldehyde for reducing 3-hydroxy-2-oxopropanal. Additionally, Figure S7f shows that the reduction of dihydroxyacetone leads preferably to the formation of hydroxyacetone than glycerol. The subsequent reduction of hydroxyacetone can produce 1,2-propanediol and acetone, with acetone being the preferred product at higher overpotentials and 1,2-propanediol being preferred at lower overpotentials, as shown in Figure S7b. The square blocks in Figure 6 represent the products identified from CO_(2)_RR, while other intermediates, such as methylglyoxal, dihydroxyacetone, glyceraldehyde, and 1,3-propanediol, are based on the results presented in Sections S1–S5, although they were not detected in this study. If indeed 3-hydro-2-oxopropanal is formed from CO–glycolaldehyde coupling, 1,3-propanediol would not be favored on the Cu electrode since it follows the glyceraldehyde pathway. The same conclusions can be drawn for the formation of methylglyoxal from 3-hydro-2-oxopropanal. Although the hydroxyl group can be removed to form methylglyoxal, we have shown in Section S1–S5 that the aldehyde group is generally further reduced to an alcohol on a Cu electrode, which would generate preferably hydroxyacetone over methylglyoxal. It is possible that these products are formed in such low quantities that we were unable to detect them. In general, the formation of C_3_ compounds other than 1-propanol as products of CO_2_RR may be limited by unfavorable intermediates, which can explain their relatively low yields. Nevertheless, understanding their reaction pathways can be valuable for designing future experiments focusing on the synthesis of C_3_ compounds. In this regard, this study contributes on the understanding of the reaction mechanism for hydroxyacetone, acetone, and 1,2-propanediol from CO_2_RR, which has so far not been explored in detail.

## 4 Conclusions

This work has presented new insights and evidence for the formation of C_2+_ compounds from CO_(2)_RR through the systematic reduction of several functional groups on Cu electrodes. This allowed us to derive the fundamental principles of functional group reduction on Cu electrodes. Our findings indicate that the formation of ethanol does not follow the glyoxal pathway, as previously suggested but instead likely occurs via the coupling of CH_3_* and CO. Furthermore, we proposed a reaction pathway for the formation of hydroxyacetone, acetone, and 1,2-propanediol in this work. These compounds are minor products in conventional CO_2_ electrolysis, and their formation could only be detected when long-term electrolysis was carried out. Our investigations reveal that hydroxycarbonyl compounds, such as the hydroxyacetone molecule, undergo further reduction on a Cu electrode in neutral pH to produce 1,2-propanediol and acetone. Notably, the reduction of the carbonyl group to form 1,2-propanediol is preferred over the dehydroxylation of the hydroxyl group to form acetone. This finding suggests that 1,2-propanediol and acetone follow the hydroxyacetone pathway during CO_2_RR. As hydroxyacetone is a minor product, the formation of acetone and 1,2-propanediol is also minor since they depend on the formation of hydroxyacetone. This observation is consistent with the results from CO_(2)_RR presented in this work. In turn, hydroxyacetone was found be to be likely formed through the coupling of CO and a C_2_-hydroxycarbonyl intermediate, such as a glycolaldehyde-like compound. The addition of glycolaldehyde to the CO_(2)_-saturated electrolyte indeed promotes the formation of hydroxyacetone, 1,2-propanediol, and acetone. This observation supports the hypothesis that glycolaldehyde serves as an intermediate in the formation of hydroxyacetone. The formation of glycolaldehyde during CO_2_RR is limited, which in turn limits the production of hydroxyacetone. In general, the formation of a specific C_3_ compound is dependent on the availability of the key C_2_-intermediate precursor. For instance, 1-propanol may be more efficiently produced than hydroxyacetone since the formation of the methylcarbonyl, required for 1-propanol synthesis, is a common intermediate on a Cu electrode, while the formation of hydroxycarbonyl (a glycolaldehyde-like intermediate) appears to be less common.

## Supporting Information Available

Trends of electroreduction of aldehydes, alcohols, ketones, ketonealdehydes, and hydroxycarbonyl on Cu electrodes in neutral pH and results from ^1^H-NMR and CORR with interval addition of glycolaldehyde in 0.1 M KOH electrolyte, respectively (PDF)
