# Supplementary material for: Mechanistic Insights into the Formation of Hydroxyacetone, Acetone, and 1,2-Propanediol from Electrochemical CO2 Reduction on Copper
Source: J Am Chem Soc. 2023 Jul 10;145(28):15343–52. doi: 10.1021/jacs.3c03045 (PMC10360152; doi:10.1021/jacs.3c03045)
Supplement: Supplementary file 1 — ja3c03045_si_001.pdf [file ja3c03045_si_001.pdf]

Supporting Information for:

Mechanistic insights into the formation of hydroxyacetone, acetone, and 1,2-propanediol from electrochemical CO_2 reduction on copper

Alisson H. M. da Silva, Georgios Karaiskakis, Rafaël E. Vos, Marc T. M. Koper*

Leiden Institute of Chemistry, Leiden University, Leiden, Netherlands.

AUTHOR INFORMATION

Corresponding author

Marc T.M. Koper – Leiden Institute of Chemistry, Leiden University, 2300 RA Leiden, The Netherlands. ORCID ID: https://orcid.org/0000-0001-6777-4594 ,

E-mail: m.koper@lic.leidenuniv.nl

Authors:

Alisson H. M. da Silva – Leiden Institute of Chemistry, Leiden University, 2300 RA Leiden, The Netherlands; ORCID ID: https://orcid.org/0000-0003-3198-3110

Georgios Karaiskakis – Leiden Institute of Chemistry, Leiden University, 2300 RA Leiden, The Netherlands

Rafaël E. Vos – Leiden Institute of Chemistry, Leiden University, 2300 RA Leiden, The Netherlands; ORCID ID: https://orcid.org/0000-0003-1810-1179

S1. Electroreduction of aldehydes

Aldehydes can be easily reduced to the corresponding alcohol in a two-electron transfer step. Furthermore, aldehyde groups are quite reactive and the pH must be controlled to prevent misinterpretation due to purely chemical reactions such as aldol condensation[1]. Therefore, to better understand the electroreduction of aldehydes on a Cu electrode, a strongly buffered neutral electrolyte must be used to prevent dimerization reactions in alkaline solutions or non-electrochemical hydrogenation to alcohols in strongly acidic media[2]. Here, we investigated how monoaldehydes and dialdehydes behave under reducing potentials on a Cu electrode. To avoid purely chemical reactions during the electroreduction, the reduction was carried out in potassium phosphate buffer (0.1 M, pH 7). For monoaldehydes, a mixture of formaldehyde, acetaldehyde, and propionaldehyde (15 mM each) was reduced between –0.7 V to –1.1 V until 15 C of charge transferred was reached. The results are shown in Figure S1. The liquid products identified were only their corresponding alcohol: methanol, ethanol, and 1-propanol. For gaseous products, H_2 was the only compound detected due to the hydrogen evolution, a parallel reaction that cannot be avoided in a water-based electrolyte. Importantly, methane, ethane or propane were not detected in any test, indicating that the oxygen atom in the carbonyl is not easily removed from the aldehyde to form a hydrocarbon molecule at neutral pH on Cu electrode. C-C coupling between the aldehydes did not take place since no C_{4+} compounds (such as crotonaldehyde) were observed in these tests and, therefore, aldol condensation was prevented by using the buffer electrolyte. An optimum to alcohols formation was found at –0.8 V. Potentials more negative than –0.8 V resulted in higher FE to H_2, which was likely promoted by the phosphate anions that act as proton donor[3].

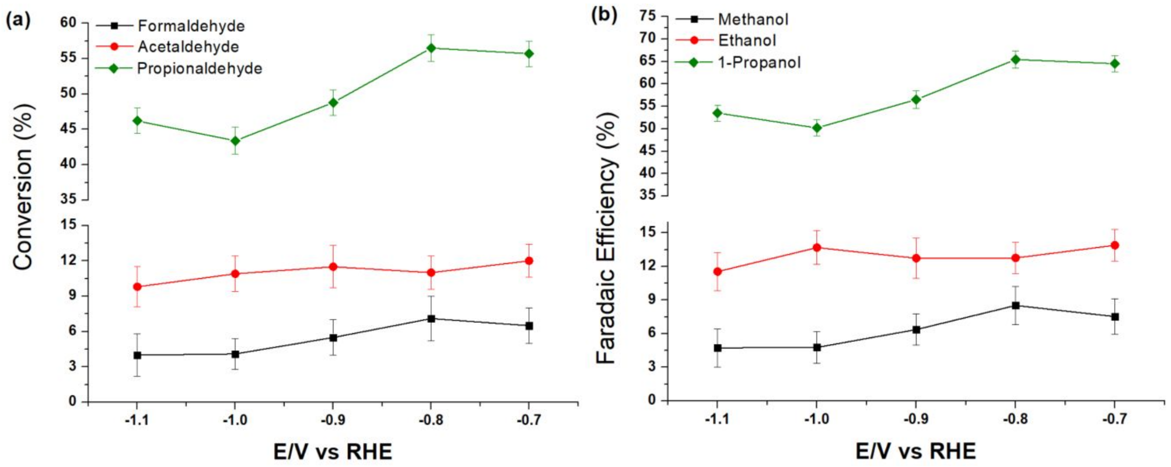

Figure S1. (a) Conversion of formaldehyde (black line), acetaldehyde (red line), and propionaldehyde (green line) and (b) corresponding Faradaic efficiencies for methanol (black line), ethanol (red line), and 1-propanol (green line) obtained after 15 C of charge was transferred. Ar-saturated 0.1 M potassium phosphate buffer (pH 7) with 15 mM of each aldehyde was used as electrolyte.

There is a positive correlation between the carbon chain length of the aldehyde molecule and its conversion and faradaic efficiency (FE) (i.e. propionaldehyde > acetaldehyde > formaldehyde). To investigate whether the mixture of formaldehyde, acetaldehyde, and propionaldehyde is somehow inhibiting the conversion of formaldehyde, the reduction of each aldehyde was carried out individually. The same trend for aldehyde conversion was observed in all potentials: propionaldehyde > acetaldehyde > formaldehyde. Propionaldehyde is more reactive than formaldehyde and acetaldehyde because the longer chain leads to a higher electron density on the carbonyl group, turning it more reactive[4].

The electroreduction of formaldehyde, acetaldehyde, and propionaldehyde on a Cu electrode was also investigated in a CO_2-saturated $KHCO_3$ electrolyte (0.1 M, pH 6.8) to determine if the observed trend in the buffer electrolyte was consistent with the conventional CO_2 reduction conditions. The same trend was observed for all three aldehydes (Fig. S2). Lower conversions for aldehydes were detected due to the

competition with CO_2RR on Cu surface[5]. As observed in phosphate electrolyte, no ethane or propane was detected between –0.7 V and –1.1 V. Formation of methane was found to be lower than 1% and similar to that observed when CO_2 was reduced in the absence of aldehydes in solution. Therefore, the detected methane was likely formed through CO_2 reduction only.

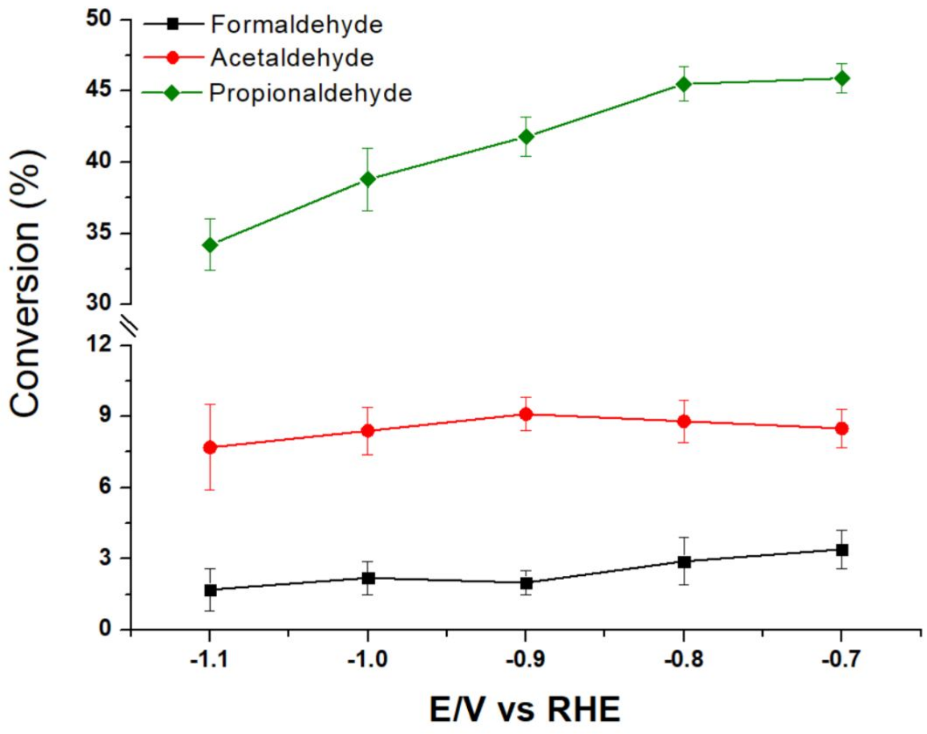

Figure S2. Conversion of formaldehyde (black line), acetaldehyde (red line), and propionaldehyde (green line) obtained after 15 C of charge was transferred. 0.1 M CO_2-saturated $KHCO_3$ (pH = 6.8) with 15 mM of each aldehyde was used as electrolyte.

Based on the results obtained from the reduction of monoaldehydes, which showed that the oxygen atom in the carbonyl group is not removed and the only product formed is the corresponding alcohol, it is reasonable to expect that the reduction of dialdehydes would result in the formation of the corresponding hydroxyaldehyde and diol products. To check this hypothesis, we investigated the reduction of glyoxal under the same conditions as above; the results are shown in Figure S3. The main products formed were glycolaldehyde and ethylene glycol, which

are the expected hydroxyaldehyde and diol products, respectively. We observed that the formation of ethylene glycol increased with the overpotential, as the second aldehyde group in the glycolaldehyde molecule was further reduced to its respective alcohol. However, at potentials more negative than –0.8V, acetaldehyde and ethanol were also detected. This suggests that, unlike monoaldehydes, one of the oxygen atoms from the carbonyl group in the dialdehyde molecule could be removed to form the monoaldehyde and subsequently the monoalcohol compounds. This can be explained by the presence of carbonyl group adjacent to the hydroxyl group, which makes the later susceptible to further reduction. Further results on the reduction of hydroxyaldehydes and hydroxyketones are provided in section S5. We did not observe the formation of hydrocarbons such as ethane, which confirms that only one oxygen atom from the carbonyl group in the dialdehyde can be removed to form a monoaldehyde while the second carbonyl or alcohol group remains intact.

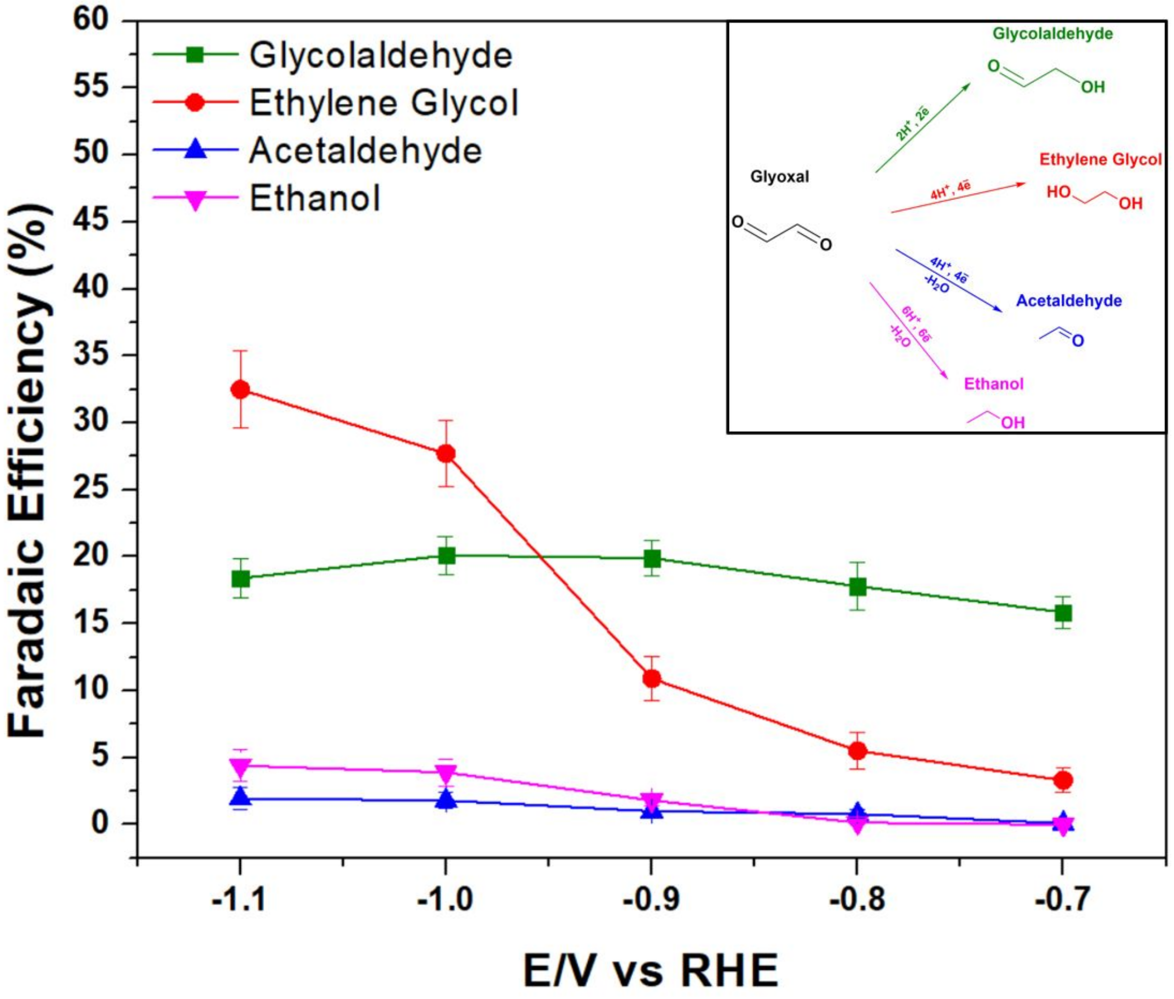

Figure S3. Faradaic efficiencies for the reduction of glyoxal towards glycolaldehyde (green line), ethylene glycol (red line), acetaldehyde (blue line), and ethanol (pink line) obtained after 15 C of charge was transferred. 0.1 M potassium phosphate buffer (pH = 7) with 25 mM of glyoxal was used as electrolyte.

The reduction of mono- and dialdehydes on a Cu electrode is useful in helping to understand the reaction mechanism of C_{2+} compounds from CO_2RR, which will be discussed in the results of the main text. In general, based on the results presented here, if an aldehyde is considered an intermediate, we can consider that a monoaldehyde is only electrochemically reduced to its corresponding alcohol, and a dialdehyde can be reduced to its respective hydroxyaldehyde and diol but can also be further reduced to its monoaldehyde and monoalcohol.

S2. Electroreduction of alcohols

Unlike aldehydes, alcohols are more stable final products of CO_2RR and CORR. This is supported by the fact that ethanol and 1-propanol can achieve higher faradaic efficiencies from CO_2 or CORR on Cu-based electrodes[6–10], while the same is not observed for acetaldehyde and propionaldehyde. This suggests that the hydroxyl group in alcohol molecules is not easily removed to form hydrocarbons. If the hydroxyl group in the alcohol molecules were easily removed to form hydrocarbons, it would be difficult to stop the reaction at a point where alcohols are preferred over hydrocarbons, and therefore the reported higher faradaic efficiencies for ethanol and 1-propanol would not be possible. To confirm this hypothesis, a systematic study was conducted to evaluate the effect of the number and position of hydroxyl groups in the carbon chain under negative potentials. For this, we evaluated the reduction of methanol, ethanol, 1-propanol, 2-propanol, ethylene glycol, 1,2-propanediol, 1,3-propanediol, and glycerol. Our studies started with the reduction of monoalcohols. 50mM of methanol, ethanol, 1-propanol, or 2-propanol were reduced in potassium phosphate buffer (0.1M, pH 7). Only H_2 was identified as product in all cases. To confirm this trend in a conventional CO_2RR conditions, the alcohols were also reduced in CO_2-sautrated $KHCO_3$ (0.1M, pH 6.8) electrolyte, and the concentration before and after did not change (for methanol and 2-propanol) or slightly increased (for ethanol and 1-propanol) after 15C was transferred due to the formation of these alcohols from CO_2RR. The reduction of diols and triols was also performed under the same conditions. 50 mM of ethylene glycol, 1,2-propanediol, 1,3-propanediol or glycerol were reduced in phosphate buffer electrolyte and, once again, we did not see any conversion of these alcohols and only H_2 was detected as product. Thus, regardless of the number of carbons in the carbon chain, the position of the hydroxyl (in the case

for 1-propanol *vs*. 2-propanol or 1,2-propanediol *vs.* 1,3-propanediol) or the number of hydroxyls in the molecule (1-propanol or 2-propanol *vs.* 1,2-propanediol or glycerol), alcohols are not reduced at neutral pH on a Cu electrode.

S3. Electroreduction of ketones

Acetone is the simplest and smallest ketone molecule, and like aldehydes, it can undergo aldol condensation in alkaline conditions[1]. Therefore, in order to investigate its electroreduction, the control of pH is an important parameter to prevent dimerization. To this end, 50 mM of acetone was reduced in a 0.1 M potassium phosphate buffer at pH 7. Surprisingly, acetone was almost inactive, with less than 1% conversion observed at all potentials (Fig.S4a). 2-propanol was the only observed product, and propane was not found in the detection limit of the gas-chromatograph. The only gas identified in the reaction was H_2. To confirm this trend under conventional CO_2RR conditions, we also carried out the reaction in CO_2-saturated $KHCO_3$ electrolyte and obtained the same results. Acetone reduction is commonly reported on a Pt electrode in acidic media and 2-propanol and propane are commonly detected as outcomes[11–14]. The pH has an important role for the carbonyl reduction[4]. To check the reactivity of acetone on Cu in acidic media, 50 mM acetone was reduced in 0.1M $HClO_4$. A higher concentration of 2-propanol was observed in comparison to neutral pH (Fig.S4b) but propane was not detected. Therefore, on Cu, as observed for aldehydes, the carbonyl group can only be reduced to hydroxyl (but relatively small conversion) but not to hydrocarbons. Differently from Pt, propane was not observed, showing that besides the pH, the nature of the metal is also an important parameter.

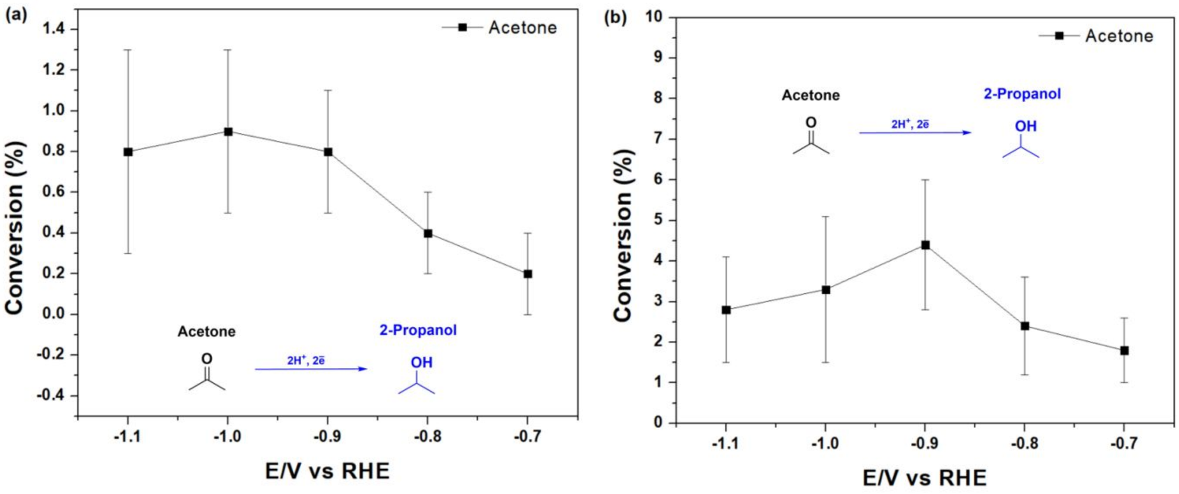

Figure S4. Conversion of acetone obtained after 15C of charge was transferred in (a) 0.1 M potassium phosphate buffer (pH 7) with 25 mM of acetone as electrolyte and (b) 0.1M $HClO_4$ (pH 1).

It is worth noting that the position of the carbonyl plays a significant role. Propionaldehyde, a C_3 compound with the carbonyl located on the first carbon (aldehyde group), exhibits high reactivity even at neutral pH. However, under the same conditions, acetone, a C_3 compound with the carbonyl on the second carbon (ketone group), shows minimal reactivity. Even at more acidic electrolyte, acetone conversion is smaller than propionaldehyde in neutral pH. The reduction of these two molecules, mixed in a buffer solution (25 mM each), shows that 1-propanol is formed while not even traces of 2-propanol are detected (Fig. S5). Thus, for the CO_2RR mechanism, the position of the carbonyl is an essential factor, where aldehydes will reduce easier than ketones. This is because the aldehyde functional group (CHO) is a more reactive functional group than the ketone functional group (C=O) as generally found in organic chemistry. This is due to a combination of steric hinderance and electronic effects[4]. The aldehyde functional group is more electrophilic than the ketone functional group, meaning it is more likely to attract nucleophiles and undergo reactions such as nucleophilic addition or reduction[4].

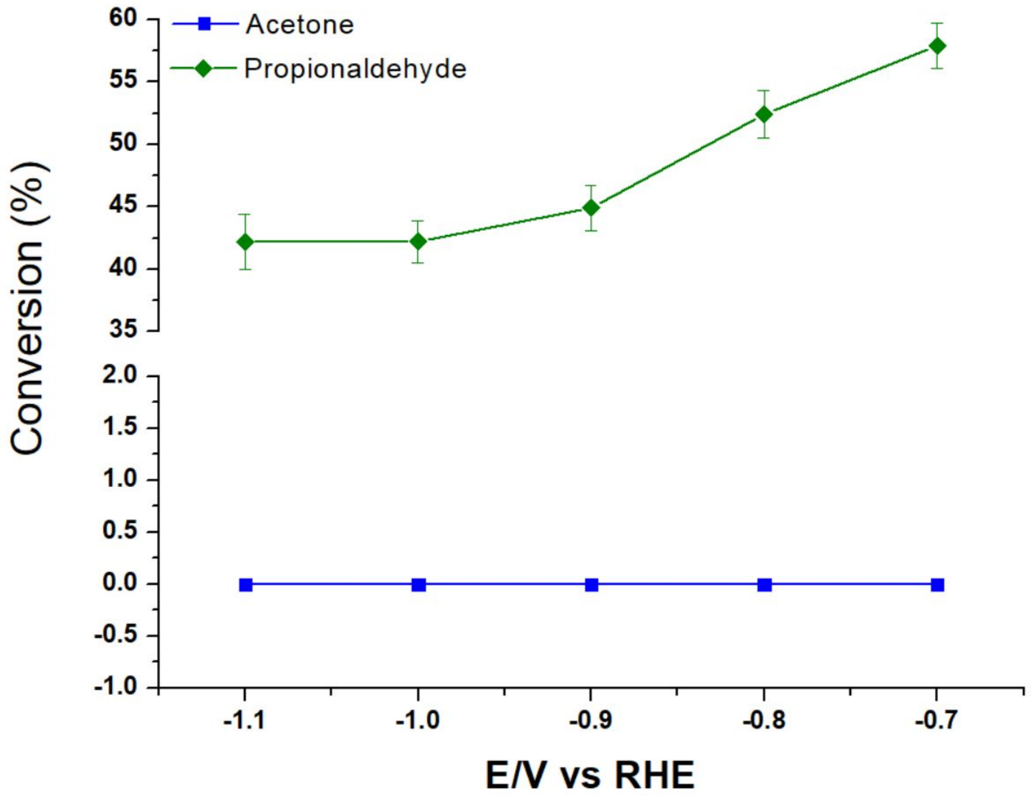

Figure S5. Conversion of propionaldehyde (green line) and acetone (blue line obtained after 15C of charge was transferred. 0.1 M potassium phosphate buffer (pH 7) with 25 mM of each molecule was used as electrolyte.

Therefore, from the results we have shown here, the carbonyl reactivity depends on its position and an aldehyde would preferably react over ketone when both are mixed in the same solution. These results will be important to guide the reaction pathway for CO_2RR towards C_3 compounds.

S4. Electroreduction of ketonealdehyde

We have shown in the previous section that the reactivity of the carbonyl group is influenced by its position in the carbon chain, as exemplified by comparing the reduction of acetone and propionaldehyde. In this section, we explore a similar trend by examining the behavior of these two functional groups when both ketone and carbonyl groups are present in the same molecule. To investigate this, we used methylglyoxal as a model molecule to better understand the behavior of a ketone group and an aldehyde group when reduced on a Cu electrode. The results obtained from methylglyoxal reduction are presented in Figure S6. At the lowest overpotentials (–0.7 V and –0.8 V), hydroxyacetone and 2-hydroxypropanal were the primary compounds identified, with hydroxyacetone showing the highest FE. Based on the results from the section S3, the aldehyde group located on the first carbon is more reactive than the ketone group located on the second carbon, thus favoring the formation of hydroxyacetone over 2-hydroxypropanal. However, in contrast to what was observed during acetone reduction, the presence of an adjacent aldehyde group significantly enhances the reactivity of the ketone group. While the reduction activity of the ketone to form 2-hydroxypropanal in the methylglyoxal molecule is a bit lower than the reactivity of the aldehyde group to form hydroxyacetone (FE of 15.5% for 2-hydroxyacetone vs. 18.5% for hydroxyacetone at –0.7 V), the formation of 2-propanol during acetone reduction is negligible, reinforcing the enhancement of the reactivity of the ketone group when it is placed adjacent to another carbonyl. When a higher overpotential is applied, 1,2-propanediol, propionaldehyde, 1-propanol, and acetone are detected. The formation of 1,2-propanediol occurs due to the further reduction of hydroxyacetone and 2-hydroxypropanal via a two-electron transfer step. The

formation of acetone, propionaldehyde, and 1-propanol will be discussed in the subsequent section regarding hydroxyketones and hydroxyaldehydes.

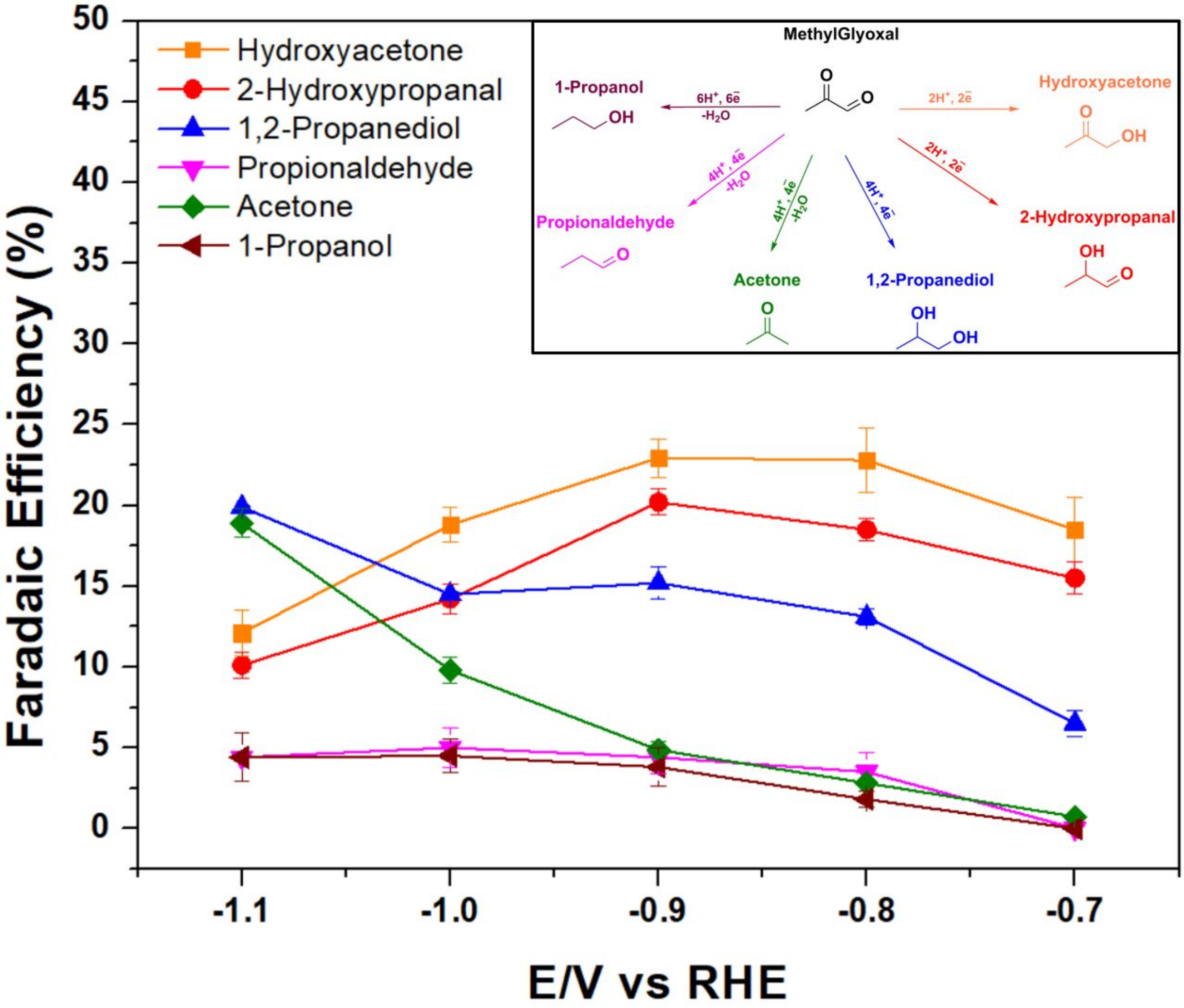

Figure S6. Faradaic efficiencies for hydroxyacetone (orange line), 2-hydroxypropanal (red line), 1,2-propanediol (blue line), propionaldehyde (pink line), acetone (green line), and 1-propanol (brown line) obtained after 15C of charge transferred. 0.1M potassium phosphate buffer (pH = 7) with 25 mM methylglyoxal was used as electrolyte.

To summarize, considering the results presented in sections S3 and S4, it is evident that the carbonyl in the first carbon (aldehyde group) exhibits higher reactivity than the carbonyl in the second carbon (ketone group), but the presence of an adjacent carbonyl enhances the reactivity of the ketone group.

S5. Electroreduction of hydroxycarbonyl

In sections S1 and S4, we demonstrated that the reduction of a dialdehyde or a ketonealdehyde produces hydroxycarbonyls, including hydroxyaldehydes like glycolaldehyde and 2-hydroxypropanal, or hydroxyketones such as hydroxyacetone, which can be further reduced to their respective diols. Additionally, we found that one of the oxygen atoms could be removed from hydroxycarbonyls resulting in the formation of their respective aldehyde or ketone, as observed in the reduction of glyoxal, which led to the formation of acetaldehyde and ethanol at higher overpotentials, and in the reduction of methylglyoxal, which led to the formation of propionaldehyde, 1-propanol, and acetone. In this section, we systematically investigate the behavior of the hydroxyl group in the presence of a carbonyl group adjacent to it. To achieve this, we investigated the reduction of glycolaldehyde (Fig.S7a), hydroxyacetone (Fig.S7b), 2-hydroxypropanal (Fig.S7c), 3-hydroxypropanal (Fig.S7d), glyceraldehyde (Fig.S7e), and dihydroxyacetone (Fig.S7f), which allows us to assess the impact of the position and number of hydroxyl groups in relation to the carbonyl group in the carbon chain. Specifically, we will investigate what happens with the hydroxyl group: 1) when the hydroxyl is placed in the first carbon while the carbonyl is adjacent (glycolaldehyde and hydroxyacetone); 2) when the hydroxyl is in the second carbon while the carbonyl is adjacent (2-hydroxypropanal); 3) when the hydroxyl is in the third carbon and the carbonyl in the first carbon (3-hydroxypropanal); 4) when two hydroxyls are placed in the second and third carbon while the carbonyl is placed in the first one (glyceraldehyde); and 5) when two hydroxyls are placed in the first and third carbon while the carbonyl is placed in the second one (dihydroxyacetone). Our results indicate that reduction of the carbonyl group is the most favored reaction at lower overpotentials (–0.7 V and –0.8 V) in all

cases, except for dihydroxyacetone. Diols and triols were the primary products observed, including ethylene glycol, 1,2-propanediol, 1,3-propanediol and glycerol. At potentials more negative than –0.8 V, it is more evident that the hydroxyl group adjacent to the carbonyl group can be removed: the hydroxyl from glycolaldehyde was removed to form acetaldehyde (Fig.S7a); similarly, hydroxyl group was removed from hydroxyacetone and 2-hydroxypropanal to form acetone (Fig.S7b) and propionaldehyde (Fig.S7c), respectively. The phenomenon of removing hydroxyl groups next to the carbonyl is confirmed by the reduction of dihydroxyacetone. One of the hydroxyls was selectively removed to form hydroxyacetone (Fig.S7f), but acetone was also detected at more negative potentials, indicating that both hydroxyl groups adjacent to the carbonyl group can be removed. However, if the hydroxyl group is not adjacent to the carbonyl group, it remains unchanged. When 3-hydroxypropanal was reduced, only 1,3-propanediol was formed (Fig.S7d) while no propionaldehyde was identified. 3-hydroxypropanal was formed in the reduction of glyceraldehyde (Fig.S7e), but 2-hydroxypropanal was not detected.

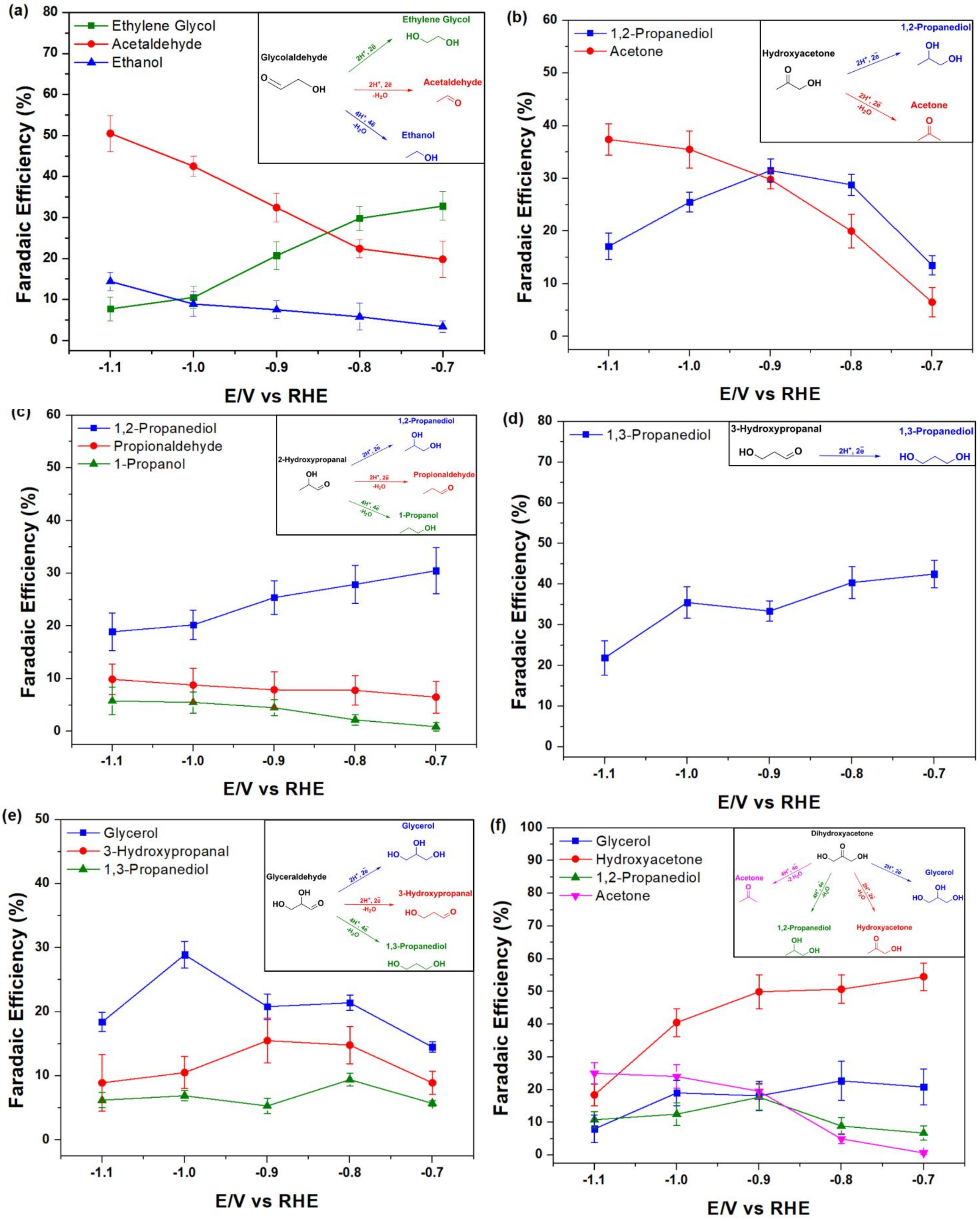

Figure S7. Faradaic efficiencies for their respective alcohols or hydroxycarbonyls when 25 mM of (a) Glycolaldehyde; (b) Hydroxyacetone; (c) 2-hydroxypropanal; (d) 3-hydroxypropanal; (e) Glyceraldehyde; or (f) Dihydroxyacetone is reduced in 0.1M potassium phosphate buffer (pH = 7).

Our group has recently investigated the reduction of dihydroxyacetone and hydroxyacetone on Pd electrodes[15,16]. Acetone is preferably formed over 1,2-propanediol at pH > 3. When glyceraldehyde was reduced, 1,3-propanediol was the preferred product. In other words, hydroxycarbonyl molecules undergo preferably dehydroxylation over carbonyl reduction to alcohols on Pd electrodes while the opposite trend is observed for Cu electrodes. These results show that the nature of the electrode is also an important parameter in the reaction pathway towards a specific product.

With the results shown in the sections S4 and S5, we can conclude that a hydroxyl group can be cleaved in from the carbon chain on Cu electrode and at neutral pH when the hydroxyl is adjacent to a carbonyl group.

Note 1. ^{1}H-NMR analysis

Figure S8 shows ^{1}H-NMR spectrum for a standard solution of 1,2-propanediol (black line) and the liquid sample from $CO_{(2)}$RR showed in Figure 4a in the main text. The signals observed for a standard solution of 1,2-propanediol centered at 3.6 and 3.5 ppm result from the diastereomeric methylene protons (H^3 and $H^{3'}$, CH_2 group) split by the neighboring methine proton (H^2, CH group) and each other, resulting in the doublet of doublets[17,18]. These peaks are also present in the liquid sample collected from CO_2RR (red line), confirming the formation of 1,2-propanediol under $CO_{(2)}$RR conditions.

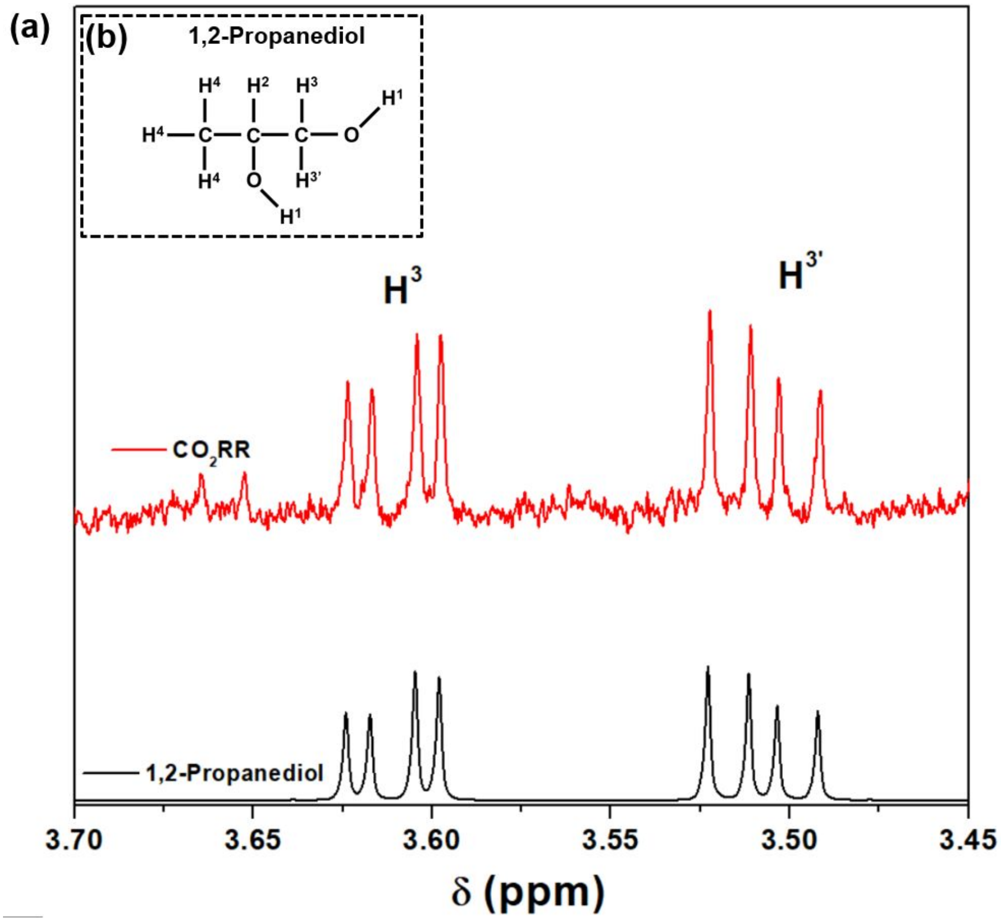

Figure S8. (a) ^{1}H-NMR spectra of 1,2-propanediol standard (black line) and liquid product from $CO_{(2)}$RR in D_2O; (b) 1,2-propanediol molecule with hydrogens atoms numbered.

Note 2. CORR with interval addition of glycolaldehyde in 0.1M KOH electrolyte

In order to enhance the formation of dehydrogenated glycolaldehyde and increase the production of C_3 minor products, CORR was carried out in 0.1M KOH with interval addition of glycolaldehyde to the electrolyte and the results are shown Figure S9. The following strategy was used: 1) the copper electrode was immersed in a solution containing 1M glycolaldehyde for 1 minute; 2) the electrode was then air-dried for 3 minutes; 3) CORR was performed at –1.0 V for 5 minutes in 0.1M KOH (pH = 13) using the Cu electrode dipped in glycolaldehyde solution. This process was repeated 18 times (90 min of electrolysis), with liquid samples collected every 30 minutes, acidified and readily injected into the chromatograph. To verify whether the products ceased forming without glycolaldehyde, the CORR was conducted uninterruptedly for an additional 30 minutes after the 18th time. Steps 1 to 3 were then reinitialized and repeated for six more times. The use of an alkaline electrolyte was intended to enhance glycolaldehyde dehydrogenation. Since glycolaldehyde undergoes aldol condensation rapidly, it was not directly added to the electrolyte. The aim of this strategy was to enhance the concentration of dehydrogenated glycolaldehyde on the Cu surface, although C_{4+} compounds from aldol condensation were still detected in solution. Figure S9b show the chromatogram recorded after the samples was collected and analyzed, depicting the upward trend of hydroxyacetone and 1,2-propanediol over time.

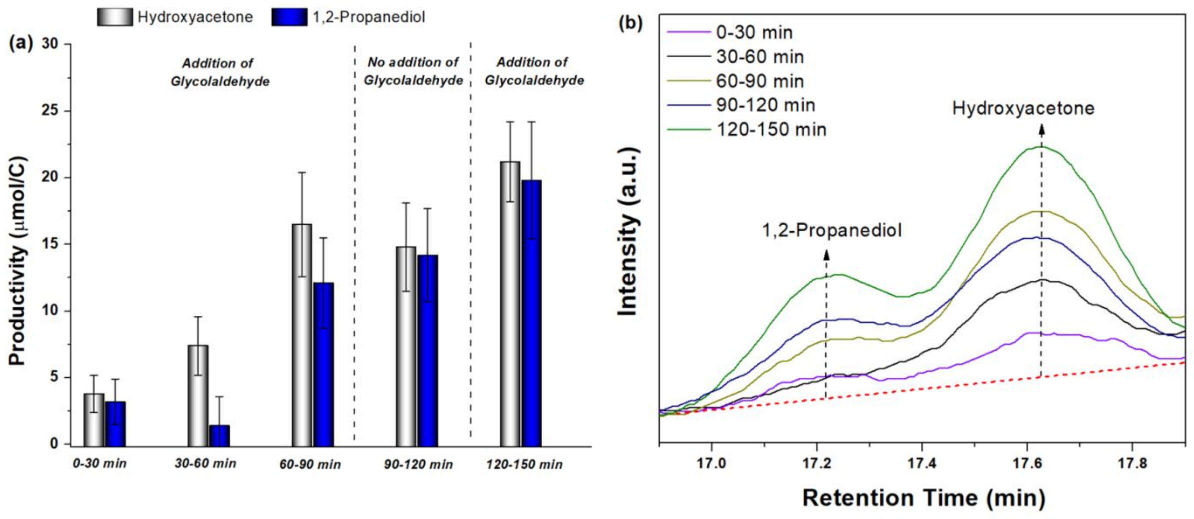

Figure S9. (a) Productivity for hydroxyacetone (grey bar) and 1,2-propanediol (blue bar) in 0.1 M KOH following the steps described in the Note 2. (b) Chromatogram of the liquid samples analyzed every 30 minutes of reaction.

References

1. Nielsen, A. T. & Houlihan, W. J. The Aldol Condensation. in *Organic Reactions* 1–438 (John Wiley & Sons, Inc., 2011). doi:10.1002/0471264180.or016.01.
2. Doyle, M. P. *et al.* Silane reductions in acidic media. III. Reductions of aldehydes and ketones to alcohols and alcohol derivatives. General syntheses of alcohols, symmetrical ethers, carboxylate esters and acetamides. *J Org Chem* **39**, 2740–2747 (1974).
3. Jackson, M. N., Jung, O., Lamotte, H. C. & Surendranath, Y. Donor-Dependent Promotion of Interfacial Proton-Coupled Electron Transfer in Aqueous Electrocatalysis. *ACS Catal* **9**, 3737–3743 (2019).
4. Clayden, J., Greeves, N. & Warren, S. Nucleophilic addition to the carbonyl group. in *ORGANIC CHEMISTRY* (eds. Clayden, J., Greeves, N. & Warren, S.) 125–140 (OXFORD, 2012).
5. da Silva, A. H. M., Lenne, Q., Vos, R. E. & Koper, M. T. M. Competition of CO and Acetaldehyde Adsorption and Reduction on Copper Electrodes and Its Impact on *n* -Propanol Formation. *ACS Catal* 4339–4347 (2023) doi:10.1021/acscatal.3c00190.
6. Kwon, Y., Lum, Y., Clark, E. L., Ager, J. W. & Bell, A. T. CO_2 Electroreduction with Enhanced Ethylene and Ethanol Selectivity by Nanostructuring Polycrystalline Copper. *ChemElectroChem* **3**, 1012–1019 (2016).
7. Ding, L. *et al.* Over 70 % Faradaic Efficiency for CO_2 Electroreduction to Ethanol Enabled by Potassium Dopant-Tuned Interaction between Copper Sites and Intermediates. *Angewandte Chemie International Edition* **61**, (2022).
8. Rudd, J. A. *et al.* Investigation into the Re-Arrangement of Copper Foams Pre- and Post-CO2 Electrocatalysis. *Chemistry (Easton)* **3**, 687–703 (2021).
9. Wu, G. *et al.* Selective Electroreduction of CO_2 to *n* -Propanol in Two-Step Tandem Catalytic System. *Adv Energy Mater* **12**, 2202054 (2022).
10. Pang, Y. *et al.* Efficient electrocatalytic conversion of carbon monoxide to propanol using fragmented copper. *Nat Catal* **2**, 251–258 (2019).
11. Bondue, C. J. & Koper, M. T. M. A mechanistic investigation on the electrocatalytic reduction of aliphatic ketones at platinum. *J Catal* **369**, 302–311 (2019).
12. Bänsch, B., Härtung, Th., Baltruschat, H. & Heitbaum, J. Reduction and oxidation of adsorbed acetone at platinum electrodes studied by DEMS. *J Electroanal Chem Interfacial Electrochem* **259**, 207–215 (1989).
13. de Hemptinne, X. & Schunck, K. Electrochemical reduction of acetone. Electrocatalytic activity of platinized platinum. *Transactions of the Faraday Society* **65**, 591 (1969).

14. Bondue, C. J., Calle-Vallejo, F., Figueiredo, M. C. & Koper, M. T. M. Structural principles to steer the selectivity of the electrocatalytic reduction of aliphatic ketones on platinum. *Nat Catal* **2**, 243–250 (2019).

15. Liang, Z. *et al.* Electrochemical Reduction of the Simplest Monosaccharides: Dihydroxyacetone and Glyceraldehyde. *ACS Catal* **10**, 13895–13903 (2020).

16. Liang, Z., Villalba, M. A. & Koper, M. T. M. Structure sensitivity of electrochemical adsorption and reduction of acetol on noble metal electrodes. *Electrochim Acta* **391**, 138911 (2021).

17. Komoroski, E. M., Komoroski, R. A., Valentine, J. L., Pearce, J. M. & Kearns, G. L. The Use of Nuclear Magnetic Resonance Spectroscopy in the Detection of Drug Intoxication. *J Anal Toxicol* **24**, 180–187 (2000).

18. Dauner, B. R. & Pringle, D. L. Proton NMR Analysis of Heat Exchange Fluids Containing Ethylene Glycol, Propylene Glycol, and Water: A Real-World Experiment for the Analytical Laboratory. *J Chem Educ* **91**, 743–746 (2014).
